# Supplementary material for: Insurance claims data: a possible solution for a national sports injury surveillance system? An evaluation of data information against ASIDD and consensus statements on sports injury surveillance
Source: BMJ Open. 2014 Jun 12;4(6):e005056. doi: 10.1136/bmjopen-2014-005056 (PMC4067892; doi:10.1136/bmjopen-2014-005056)
Supplement: Author's manuscript [file bmjopen-2014-005056.draft_revisions.pdf]

Sports injury surveillance at a national level; evaluation of insurance claims data

Journal:	*BMJ Open*
Manuscript ID:	bmjopen-2014-005056
Article Type:	Research
Date Submitted by the Author:	14-Feb-2014
Complete List of Authors:	Åman, Malin; The Swedish School of Sport and Health Scienses, Performance and training<br>Forssblad, Magnus; Department of Molecular Medicine and Surgery, Karolinska Institut, Stockholm Sports Trauma Research Center, Capio Artro Clinic<br>Henriksson-Larsén, Karin; The Swedish School of Sport and Health Scienses, Principal
**Primary Subject Heading**:	Sports and exercise medicine
Secondary Subject Heading:	Sports and exercise medicine
Keywords:	SPORTS MEDICINE, EPIDEMIOLOGY, PREVENTIVE MEDICINE, PUBLIC HEALTH

SCHOLARONE™ Manuscripts

For peer review only

Title

Sports injury surveillance at a national level; evaluation of insurance claims data

Corresponding author

Malin Åman
GIH -The Swedish School of Sport and Health and Sciences
Lidingövägen 1, Box 5626, 114 86 Stockholm, Sweden.
malin.aman@gih.se
telephone +46739947349, fax +46812053880

Co-authors

Magnus Forssblad
Department of Molecular Medicine and Surgery, Karolinska Institut,
Stockholm Sports Trauma Research Center, Capio Artro Clinic,
Box 5605, 114 86 Stockholm, Sweden

Karin Henriksson-Larsén
GIH -The Swedish School of Sport and Health and Sciences
Lidingövägen 1, Box 5626, 114 86 Stockholm, Sweden.

Keywords

Sports injury, athletic injury, accident prevention, recreation, epidemiology

Word count

2 792

Number of references

33

For peer review only

ABSTRACT

Background:

Before preventive actions can be suggested for sports injuries at a national level, epidemiology, risk factors and mechanisms need to be inventoried and there are guidelines for sport injury data collection and classifications in the literature. In Sweden 90% of all athletes and 57 sport federation are insured in the same insurance company.

Objective:

To evaluate the usefulness of sports injury insurance claims in sports injury surveillance, at a national level.

Method:

An Excel file with 27947 injuries, as well as access to the database for text information was used. Data were reviewed on available information, missing information, and drop-outs. Comparison against ASIDD (Australian Sports Injury Data Dictionary) and with existing Consensus statement in the literature (Soccer, Rugby, Tennis, Cricket and Thoroughbred horse racing) was performed in a structured manner.

Result:

Comparison with ASIDD showed that 93 % of the suggested data items were present in the database, to at least some extent. Compliance with the Consensus statements was generally high (13/18). Almost all claims (83%) contained text information surrounding the injury.

Conclusion:

Relatively high quality sports injury data can be obtained from a specific insurance company in Sweden. The database has the potential to be a solid base for research on acute sport injuries in different sports at a national level.

For peer review only

Strengths and limitations of this study:

- It includes a large material from a database that cowers more than 80% of the sports federations in Sweden at a national level.
- It is a structured examination of the reliability and validity of data, using standardized guidelines from the sport injury literature.
- A limitation is that data is not evaluated for recording rate reliability since there is no national record of sports injuries in the country.

For peer review only

INTRODUCTION

Since approximately 18-30 % of all acute injuries (accidental injuries) are sports-related, (1, 2) preventive actions are warranted. It is suggested that almost half these injuries are related to team sports, in particular soccer, handball and basketball. (2-4) This type of injury, a traumatic injury, is defined as an injury resulting from a specific, identifiable event. (2, 4) Prevention of sport injuries requires four stages: (1) identifying the magnitude of the problem (epidemiology), (2) identifying risk factors that may contribute to injuries (etiology), (3) introducing a preventive measure (prevention), and (4) assessing prevention effectiveness by repeating step 1.(5) There are guidelines for standardizations of injury definitions and data collection in the literature.(5-10) In soccer, rugby, tennis, cricket and thoroughbred horse racing there are Consensus statements on these procedures.(6, 10-13) In order to perform comparative studies it is important to have a standardized method that allows for comparison between various types of sports.

Most countries do not have a national sports injury surveillance system where injury information from several sports is found in the same database. Data for sports injury epidemiology may be obtained from emergency departments,(2, 14) insurance companies, (15-18) other records for specific sports, (19, 20) or from a specific competition. (8, 21, 22) Studies have found that data from insurance companies could be eligible in sport injury research.(16, 23, 24) Finch (2003) evaluated data from three insurance companies in Australia, compared these data with the desirable and necessary information suggested by ASIDD (The Australian Sports Injury Data Dictionary),(25) and found that relatively high quality sports injury data can be obtained from insurance claims. (23)

At the time of the present study, 57 out of 70 sport federations under The Swedish Sports Confederation (RF) use the same Swedish insurance company for injury insurance. This type of insurance covers the athlete for costs associated with their care and treatment after an accidental injury. If the injury results in a medical disability the athlete receives a lump sum in relation to the severity of the disability. In the case of death, a lump sum is paid to the family. All injuries and payments are registered in a database which covers 90% of all athletes in Sweden. Since previous studies have shown that data from insurance companies can be useful in sports injury research, (16, 23, 26) the aim of this study was to evaluate the use of the database from this particular Swedish insurance company in injury preventive research, and to compare it to ASIDD and Consensus statements on sport injuries.

For peer review only

Specific aims:

Specific aims of the study were to measure the reliability of the sports injury data by reviewing the injury data collection procedures and a sample of injuries within the database. The validity of the data was examined by comparing the included variables within the database with variables in ASIDD and Consensus statements from the literature on injury definitions and data collection procedures.(6, 10-13, 23, 25) After these evaluations, improvements to the database will be suggested.

METHODS

A sports injury can be registered with the Swedish insurance company within three years from the time the accident occurred. The definition of an injury eligible for an insurance claim is a new injury receiving medical attention. Information regarding the injury and claim is routinely kept in a systematic and structured manner within the sports injury database. From the database, details can be transformed into an Excel file for statistical analysis. The insurance company performs their own audit of the administrators' claim processing. Randomly selected injury claims, a percentage of the total number of claims, are reviewed by senior administrators every month. Once a year the company auditors also undertake an extensive review of randomly selected injury claims. The latest audit on sports injury claims was performed in the autumn of 2011, and concluded that claim settlements were generally performed with high quality and accuracy.

An Excel file containing 35 511 posts of settled injury claims between 2004 and 2010 including 27 947 unique injuries, and access to a local database for text information surrounding the injury, was received from the insurance company. Data in the Excel file included information on sports activity, age, gender, residence at time of injury, date of injury, diagnosis code (type of injury, injured body region and body part, left or right side of body), and type of financial compensation (medical treatment, dental treatment, permanent disability or death). The file also contained information on disability assessment, the administrator, and costs. The local database contained text files with descriptions of injury mechanisms, training or competition, surface and environment, and protective equipment. The local database also included information on the administrator´s procedure.

To ensure data reliability, the Excel file was reviewed for available information, missing information, and data drop-outs based on desirable and necessary information previously

For peer review only

defined in sports injury research. (6, 10-13, 25) From the Excel file, 310 random samples were chosen to evaluate data consistency within the Excel file compared to text files in the local database. The insurance company's claim and documentation procedures were studied. All administrators, all sports, and all ages of athletes were represented in the randomly selected samples. Data validity was examined both by comparing the injury data with ASIDD guidelines, in order to make comparisons between different sports (25), and it was also examined relative to Consensus statements in specific sports.(6, 10-13)

The Australian Sports Injury Data Dictionary, ASIDD (1997) was developed in Australia to provide standardized guidelines for injury data collection and classification in order to help prevent and control injury in sport and recreation. (25) ASIDD states that "*Core*" items (category 1) should be presented in all sports injury data collections, "*Strongly recommended*" items (category 2) should be included, where possible, to give detailed additional injury information and "*Recommended*" items (category 3) can provide further data pertaining to injury circumstances (Table 1). (23) Comparison of the Swedish insurance company data with ASIDD was performed and a scoring system was used to assess the amount of information in the insurance claims according to ASIDD guidelines: (23)

1 Data item fully present (should be fully coded according to ASIDD guidelines)

0 Data item totally absent (in ASIDD but not in the insurance database)

0.5 Data item partially present (some, but not all, of the details specified in ASIDD were in the database)

In order to ensure the validity of the data for specific sports, a comparison with five existing Consensus statements on injury definitions and data collection procedures in the literature (6, 10-13) was performed.

Statistics

The computer programs Microsoft office Excel 2007 and SPSS (SPSS for Windows, version 21.0) were used. Reliability of the claims and documentation procedures are expressed as a percentage by dividing the number of missing values with the number of reviewed posts. When validating the insurance data with ASIDD, a data category agreement score was calculated as the sum of the individual scored items. An overall agreement score calculated as the sum of the three data category agreements to determine the general quality of information. (23) Validation of the data using Consensus statements was based on the established

For peer review only

information criteria published in the scientific literature for soccer, rugby, tennis, cricket and thoroughbred horse racing. (6, 10-13)

RESULTS

Loss of data

Excel file

Lack of a valid social security number occurred in 103 of 27 947 posts (0,4%) in the Excel file. This was due to foreign athletes practicing their sport in Sweden without a Swedish social security number. The gender and age of these athletes can be determined from the text files in the local database. Fifty-five of 27 947 injuries (0.2%) lacked a diagnosis code but all codes could be identified when reading the texts in the database. Residence at the time of injury (postal code) was missing in approximately 23 % of the posts.

Local database (text files)

Text files in the local database were available for more than 80 % of the 310 random samples taken from the total number of claims. From 2011 onwards, text files were presented in almost all the claims. In some claims from 2007 and earlier, the texts were not always in the local database but could be ordered in hard copy format, which are not included in this study. The diagnosis codes were consistent in more than 88 % of the samples. Incorrect diagnoses were found in three samples (1%), unknown diagnoses in six samples (2%), and correct location but incorrect injury type in 13 samples (4%).

An evaluation of categories contained within the different sports was also performed. For example, the Swedish Ski Association has different categories for alpine skiing, cross country skiing, snowboarding etc. The Swedish Mixed Martial Arts Federation and the Swedish Motorsport Federation also have different categories. All samples tested were found to be in the correct sport, however consistency between the category in the Excel file and the category in the text file in the local database was approximately 83%.

Compliance to ASIDD

Comparison with ASIDD showed that 93 % (i.e. 28 of 30) of the suggested data items were present, to at least some extent. The overall agreement score was 24 out of a highest possible score of 30 (Table 1). Within “Core” ASIDD items, no missing data were found. All “strongly recommended” items were present in some form but full information was missing in four of them (Table 1). In “recommended” items, five out of eight were present but three of them did

For peer review only

not include full information (Table 1). A large part of the information regarding ASIDD items was found in the text files within the local database and not in the Excel file.

Table 1. Comparison of the insurance database with ASIDD:

**ASIDD "Core" data item**	**In the insurance data file**
Date of injury	yes: 1
Age	yes: 1
Gender	yes: 1
Activity when injured - broad areas	yes: 1
Mechanism of injury	yes: 1
Body region and body chart	yes: 1
Nature of injury	yes: 1
**Category agreement score** (maximum score = 7)	7
**ASIDD "Strongly Recommended" data item**	**In the insurance data file**
Person recording case information	yes: 1
Immediate source of injury record	yes: 1
Area of usual residence	yes: 1
Name of injury place - text	yes: 1
Place of injury - type	yes: 1
Sport and recreation places - specific	yes: 1
Activity when injured -name of sport or activity	yes: 1
Injury factors	yes: 1
Equipment used with intent to protect against injury	yes: 0.5
Narrative of mechanism of injury	yes: 1
Date of presentation	yes: 1
Treatment	yes: 0.5
Advice given to injured person	yes: 0.5
Referral	yes: 0.5
Treating person	yes: 1
**Category agreement score** (maximum score = 15)	13
**ASIDD "Recommended" data item**	**In the insurance data file**
Time of injury	no: 0
Date of injury record	yes: 1
Part of specific injury place	yes: 0.5
Phase or aspect of involvement in activity	yes: 0.5
Grade/level of play	yes: 0.5
Specific structure injured	yes: 0.5
Time of presentation	no: 0
Reason for presentation	yes: 1
**Category agreement score** (maximum score = 8)	4

Compliance with Consensus statements

Compliance with required information in the Consensus statements, published in sports-related scientific literature, were high (13 of 18 items) except for body mass, height, dominant leg/arm, position of play, and date of returning to sport after injury (Table 2). Some differences in desirable information were found among the different sport federations.

For peer review only

Information from seven items existed in the insurance database in some, but not all, claims (Table 2).

Table 2. Comparison between the information from the insurance database with required information in the Consensus statements.

Possible items	Soccer	Rugby	Tennis	Cricket	Thoroughbred horse racing	Insurance data
Age	x	x	x	x	x	x
Gender	x	x	x	x	x	x
Body mass	x	x	x		x	
Height	x	x	x		x	
Dominant leg/arm	x		x			
Standard/Level of play		x	x		x	x -
Position in play	x	x		x		
Date of onset	x	x	x	x	x	x
Injury diagnosis/Nature of injury	x	x	x	x	x	x
Injured side	x	x	x	x	x	x
Match /training	x	x	x	x	x	x-
Circumstances surrounding the injury	x	x	x	x	x	x -
Date of return to sport	x	x	x		x	
Mechanisms	x	x	x	x	x	x -
Free text (around the injury)	x		x			x -
Protective equipment					x	x -
Surface (playing ground)			x		x	x -
Treatment (medical)				x	x	x -

x - data exist in some but not in all claims

DISCUSSION

This study suggests that relatively high quality sports injury data can be obtained from a specific insurance company in Sweden, when ASIDD is used as the gold standard. Also, on comparing the data with Consensus statements from studies of sport injuries, the data from the insurance company is shown to be of relatively high quality.

In sports injury research, epidemiology and etiology are essential as a foundation for sport injury prevention.(5) Earlier studies have shown that insurance claims have the potential to provide detailed information about sports injuries, the records are routinely kept in a systematic manner, and comparisons between data can easily be made.(16, 23, 26) Different studies on sports injuries have used insurance data for different sports, such as netball, rugby and soccer.(16-18, 24) One study in Finland compared injury profiles in soccer, ice hockey, volleyball, basketball, judo and karate using national insurance register data.(27) Insurance databases have historically been invaluable for guiding road and workplace safety.(28-30) A

For peer review only

recent published study on administrative databases, including insurance databases, concluded that they could be a robust research tool. They allow for longitudinal designs, incidence calculation, and large sample size and power, even for rare events.(26)

The insurance data in this study included many of the ASIDD items; all "core" and "strongly recommended" items and more than 60% of the "recommended" items were present. Data items that, according to ASIDD, could be improved, related to treatment, referral and advice given to the athlete when injured.(25) Precise and specific information about injury location, phase or aspect of activity, equipment used and specific structure injured was missing.(25) One detail not registered was the grade or level of play, although it could occasionally be read in the text file that the athlete competed in a national championship.

The results from comparing the injury claim data with desirable information in the Consensus statements (6, 10-13) show that the insurance database could be useful in research on sport injuries. However, missing data on personal information such as height, body mass, dominant leg/arm, position of play, and return to play are important in some research. Information about return to play is often used to calculate the absence of sporting activity and hence the severity of the injury.(5) In the insurance data, injury type and level of permanent medical disability are two methods of grading injury severity. The definition of medical disability is set by "Insurance Sweden" (the industry organization for insurance companies in Sweden). http://www.svenskforsakring.se/Global/Invaliditetsintyg/Medicinsk_invaliditet_skador120911.pdf.

Van Mechelen suggests that an adequate sports injury surveillance system should be sensitive enough to answer "how many, how often, how long, and serious?".(31) If these questions are to be answered on a national level, where the magnitude of sports injury problems are investigated by comparing incidence rates between various sports, or detecting trends and changes over time, a system that can detect large numbers of cases is needed.(31) The system should be as simple and unambiguous as possible and should at least contain a universally applicable definition of sports injury, of sports participation and consensus on population at risk and time at risk.(31) The insurance data in this study are contained in a national register which covers athletes in more than 80% of all sports, and includes, for example, sports with many athletes such as soccer, ice hockey, handball, floorball, golf, material arts and motor sports. Athletes participating in some of these sports are known to be susceptible to many acute injuries.(2, 3)

For peer review only

Lack of full information within some items both compared with ASIDD and the Consensus statements suggests that the insurance data could be improved upon in the future with the collection of additional details and standardizations of data variables. Some of these changes are implemented in the present insurance database as the result of this study. For example, by using already existing headlines in the database, the terminology regarding injury mechanisms and injury circumstances have been standardized based on desirable sport injury information in the literature.(6, 10-13, 25)

Missing desirable information in the insurance data, including total number of athletes in each sport and exposure to sport, could be a weakness when performing epidemiological studies on sport injuries.(5, 32) The number of participants in each sport could be obtained from the specific sports federations, and exposure to sport might be estimated, also using information from the sports federations.

Despite the quality of the sports injury surveillance system, one will always be confronted with some sort of bias, recall bias, over- or underestimation of sports participation, incomplete responses, non-response, invalid injury description etc.(31) The insurance data collection is structured and routinely kept in a systematic manner, and the insurance company works to keep the data accurate and truthful. The database population consists of athletes who *report* an injury to the insurance company and therefore the data it contains likely underestimates the number of injuries. A study from 2005 on anterior cruciate ligament injuries in football, floorball, handball and ice hockey used the same insurance data as the present study and compared it to two medical databases to determine recording rate reliability.(18) The result showed that 74% of all injuries sustained were recorded in the insurance database.(18) Roos et al (1995) estimated that 98% of the soccer injuries were registered within seven years.(33) The amount of financial compensation for each claim from the insurance company may influence the rate of injury registration. More severe injuries that require medical treatment or may result in a permanent medical disability are more likely to be reported, as well as dental injuries where treatment is expensive. The athlete can report the injury within three years (sometimes up to ten years) of the accident, and medical disabilities are assessed in one to two years after the accident occurred. This may result in a delay of recording in the database and must be considered when analyzing the data.(33) Despite these weaknesses, the data are important for the detection of serious injuries and injury mechanisms in different sports at

For peer review only

national level. The data can also be used in longitudinal studies on injury prevention measures.

CONCLUSION

Before preventative actions can be suggested, sports injury epidemiology, risk factors and injury mechanisms need to be compiled.(5) In most countries, there is a lack of national sports injury surveillance systems and comparisons of injuries between sports are difficult to perform. Since national data from a Swedish insurance company contains most of the desirable information in sport injury research, and further improvements to the database information have been implemented, this database now has the potential to be a base for research on acute (accidental) injuries in different sports at a national level. It could also constitute a foundation for assessment of sport injury prevention at a national level.

For peer review only

Funding

This work was supported by the Folksam, a Swedish insurance company

Contributor ship statement

The corresponding author and co-authors have all substantial contributed to the research and to the final document by intellectual content, conception and design, analysis and interpretation of data as well as drafting and critical revision of the manuscript.

Data sharing statement

No additional data available.

Competing Interests

None

For peer review only

REFERENCES

1. Caine D, Caine C, Maffulli N. Incidence and distribution of pediatric sport-related injuries. *Clin J Sport Med.* 2006;**16**:500-13. PubMed PMID: 17119363. Epub 2006/11/23. eng.

2. Conn JM, Annest JL, Gilchrist J. Sports and recreation related injury episodes in the US population, 1997-99. *Inj Prev.* 2003;**9**:117-23. PubMed PMID: 12810736. Pubmed Central PMCID: 1730974.

3. (EuroSafe) EAfIPaSP. Injuries in the European Union. Summary of injury statisticsfor the years 2008-2010. 2013 (4).

4. Knowles SB, Marshall SW, Bowling JM et al. A prospective study of injury incidence among North Carolina high school athletes. *Am J Epidemiol.* 2006;**164**:1209-21. PubMed PMID: 17012366. Epub 2006/10/03. eng.

5. van Mechelen W, Hlobil H, Kemper HC. Incidence, severity, aetiology and prevention of sports injuries. A review of concepts. *Sports Med.* 1992;**14**:82-99. PubMed PMID: 1509229.

For peer review only

6. Fuller CW, Ekstrand J, Junge A et al. Consensus statement on injury definitions and data collection procedures in studies of football (soccer) injuries. *Clin J Sport Med.* 2006;**16**:97-106. PubMed PMID: 16603877.

7. Finch C. A new framework for research leading to sports injury prevention. *J Sci Med Sport*. 2006;**9**:3-9; discussion 10. PubMed PMID: 16616614. Epub 2006/04/18. eng.

8. Junge A, Engebretsen L, Alonso JM et al. Injury surveillance in multi-sport events: the International Olympic Committee approach. *Br J Sports Med.* 2008;**42**:413-21. PubMed PMID: 18390916. Epub 2008/04/09. eng.

9. Hammond LE, Lilley J, Ribbans WJ. Coding sports injury surveillance data: has version 10 of the Orchard Sports Injury Classification System improved the classification of sports medicine diagnoses? *Br J Sports Med.* 2009;**43**:498-502. PubMed PMID: 19050000. Epub 2008/12/04. eng.

10. Turner M, Fuller CW, Egan D et al. European consensus on epidemiological studies of injuries in the thoroughbred horse racing industry. Br J Sports Med. 2012;**46**:704-8. PubMed PMID: 22067282. Epub 2011/11/10. eng.

11. Fuller CW, Molloy MG, Bagate C et al. Consensus statement on injury definitions and data collection procedures for studies of injuries in rugby union. *Clin J Sport Med.* 2007;**17**:177-81. PubMed PMID: 17513907. Epub 2007/05/22. eng.

For peer review only

12. Pluim BM, Fuller CW, Batt ME et al. Consensus statement on epidemiological studies of medical conditions in tennis, April 2009. *Br J Sports Med.* 2009;**43**:893-7. PubMed PMID: 19900956. Epub 2009/11/11. eng.

13. Orchard J, Newman D, Stretch R et al. Methods for injury surveillance in international cricket. *J Sci Med Sport.* 2005;**8**:1-14. PubMed PMID: 15887896. Epub 2005/05/13. eng.

14. Nelson NG, McKenzie LB. Mountain biking-related injuries treated in emergency departments in the United States, 1994-2007. *Am J Sports Med.* 2011;**39**:404-9. PubMed PMID: 21076012. Epub 2010/11/16. eng.

15. de Loes M, Dahlstedt LJ, Thomee R. A 7-year study on risks and costs of knee injuries in male and female youth participants in 12 sports. *Scand J Med Sci Sports.* 2000;**10**:90-7. PubMed PMID: 10755279. Epub 2001/02/07. eng.

16. Otago L, Peake J. The role of insurance data in setting priorities for netball injury prevention strategies. *J Sci Med Sport.* 2007;**10**:105-9. PubMed PMID: 16844407.

17. Soderman K, Pietila T, Alfredson H et al. Anterior cruciate ligament injuries in young females playing soccer at senior levels. *Scand J Med Sci Sports.* 2002;**12**:65-8. PubMed PMID: 12121422. Epub 2002/07/18. eng.

18. Forssblad M, Weidenhielm L, Werner S. Knee surgery costs in football, floor ball, European team handball and ice hockey. Scand J Med Sci Sports. 2005 Feb;15(1):43-7. PubMed PMID: 15679571. Epub 2005/02/01. eng.

For peer review only

19.Westin M, Alricsson M, Werner S. Injury profile of competitive alpine skiers: a five-year cohort study. *Knee Surg Sports Traumatol Arthrosc.* 2012;**20**:1175-81. PubMed PMID: 22349602. Epub 2012/02/22. eng.

20.Junge A, Dvorak J. Injury surveillance in the World Football Tournaments 1998-2012. *Br J Sports Med.* 2013;**47**:782-8. PubMed PMID: 23632746. Pubmed Central PMCID: PMC3717759. Epub 2013/05/02. eng.

21. Engebretsen L, Soligard T, Steffen K et al. Sports injuries and illnesses during the London Summer Olympic Games 2012. *Br J Sports Med.* 2013;**47**:407-14. PubMed PMID: 23515712. Epub 2013/03/22. eng.

22. Engebretsen L, Steffen K, Alonso JM et al. Sports injuries and illnesses during the Winter Olympic Games 2010. *Br J Sports Med.* 2010;**44**:772-80. PubMed PMID: 20820057. Epub 2010/09/08. eng.

23. Finch C. How useful are insurance claim data for sports injury prevention purposes? *Inj Control Saf Promot.* 2003 Sep:181-3.

24. King DA, Hume PA, Milburn P et al. Rugby league injuries in New Zealand: a review of 8 years of Accident Compensation Corporation injury entitlement claims and costs. *Br J Sports Med.* 2009;**43**:595-602. PubMed PMID: 19553223. Epub 2009/06/26. eng.

For peer review only

25. Party TASIDW. Australian Sports Injury Data Dictionary. Guidelines for injury data collection and classification for the prevention and control of injury in sport and recreation. Working dokument. 1997.

26. Gavrielov-Yusim N, Friger M. Use of administrative medical databases in population-based research. J Epidemiol Community Health. 2013 Nov 18. PubMed PMID: 24248997. Epub 2013/11/20. Eng.

27. Kujala UM, Taimela S, Antti-Poika I et al. Acute injuries in soccer, ice hockey, volleyball, basketball, judo, and karate: analysis of national registry data. *BMJ.* 1995;**311**:1465-8. PubMed PMID: 8520333. Pubmed Central PMCID: PMC2543722. Epub 1995/12/02. eng.

28. Badii M, Keen D, Yu S et al. Evaluation of a comprehensive integrated workplace-based program to reduce occupational musculoskeletal injury and its associated morbidity in a large hospital. *Journal of occupational and environmental medicine / American College of Occupational and Environmental Medicine.* 2006;**48**:1159-65. PubMed PMID: 17099452. Epub 2006/11/14. eng.

29. McCall BP, Horwitz IB, Taylor OA. Occupational eye injury and risk reduction: Kentucky workers' compensation claim analysis 1994-2003. *Inj Prev.* 2009;**15**:176-82. PubMed PMID: 19494097. Epub 2009/06/06. eng.

30. Krafft M, Kullgren A, Lie A et al. The use of seat belts in cars with smart seat belt reminders--results of an observational study. *Traffic Inj Prev.* 2006;**7:**125-9. PubMed PMID: 16854706. Epub 2006/07/21. eng.

31. van Mechelen W. Sports injury surveillance systems. 'One size fits all'? *Sports Med.* 1 997;**24**:164-8. PubMed PMID: 9327530.

32. de Loes M. Exposure data. Why are they needed? *Sports Med.* 1997;**24**:172-5. PubMed PMID: 9327532. Epub 1997/11/05. eng.

33. Roos H, Ornell M, Gardsell P et al. Soccer after anterior cruciate ligament injury--an incompatible combination? A national survey of incidence and risk factors and a 7-year follow-up of 310 players. *Acta orthopaedica Scandinavica.* 1995;**66**:107-12. PubMed PMID: 7740937. Epub 1995/04/01. eng.

For peer review only

Insurance claims data - a possible solution for a national sports injury surveillance system?

Journal:	*BMJ Open*
Manuscript ID:	bmjopen-2014-005056.R1
Article Type:	Research
Date Submitted by the Author:	10-Apr-2014
Complete List of Authors:	Åman, Malin; The Swedish School of Sport and Health Scienses, Performance and training<br>Forssblad, Magnus; Department of Molecular Medicine and Surgery, Karolinska Institut, Stockholm Sports Trauma Research Center, Capio Artro Clinic<br>Henriksson-Larsén, Karin; The Swedish School of Sport and Health Scienses, Principal
**Primary Subject Heading**:	Sports and exercise medicine
Secondary Subject Heading:	Sports and exercise medicine
Keywords:	SPORTS MEDICINE, EPIDEMIOLOGY, PREVENTIVE MEDICINE, PUBLIC HEALTH

Title

Insurance claims data - a possible solution for a national sports injury surveillance system?

Corresponding author

Malin Åman
GIH -The Swedish School of Sport and Health and Sciences
Lidingövägen 1, Box 5626, 114 86 Stockholm, Sweden.
malin.aman@gih.se
telephone +46739947349, fax +46812053880

Co-authors

Magnus Forssblad
Department of Molecular Medicine and Surgery, Karolinska Institut,
Stockholm Sports Trauma Research Center, Capio Artro Clinic,
Box 5605, 114 86 Stockholm, Sweden

Karin Henriksson-Larsén
GIH -The Swedish School of Sport and Health and Sciences
Lidingövägen 1, Box 5626, 114 86 Stockholm, Sweden.

Keywords

Sports injury, athletic injury, accident prevention, recreation, epidemiology

Word count

3 172

Number of references

37

For peer review only

ABSTRACT

Background:

Before preventive actions can be suggested for sports injuries at a national level a solid surveillance system is required in order to study epidemiology, risk factors and mechanisms. There are guidelines for sport injury data collection and classifications in the literature for that purpose. In Sweden, 90% of all athletes, (57/70 sport federations) are insured in the same insurance company and the data from their database could be a foundation for studies on acute sports injuries, at a national level.

Objective:

To evaluate the usefulness of sports injury insurance claims in sports injury surveillance, at a national level.

Method:

A database with 27947 injuries was exported to an excel file. Access to the corresponding text files was also obtained. Data were reviewed on available information, missing information, and drop-outs. Comparison against ASIDD (Australian Sports Injury Data Dictionary) and with existing Consensus statement in the literature (Football (soccer), Rugby union, Tennis, Cricket and Thoroughbred horse racing) was performed in a structured manner.

Result:

Comparison with ASIDD showed that 93 % of the suggested data items were present in the database, to at least some extent. Compliance with the Consensus statements was generally high (13/18). Almost all claims (83%) contained text information concerning the injury.

Conclusion:

Relatively high quality sports injury data can be obtained from a specific insurance company at national level in Sweden. The database has the potential to be a solid base for research on acute sport injuries in different sports.

For peer review only

Strengths and limitations of this study:

- Data includes a large material from a database that covers more than 80% of the sports federations in Sweden at a national level.
- The study is a structured examination of the reliability and validity of data, using standardized guidelines from the sport injury literature.
- A limitation is that data is not evaluated for recording rate reliability since there is no national record of sports injuries in the country.

For peer review only

INTRODUCTION

Since approximately 18-30 % of all acute injuries are sports-related (1, 2), and almost half these injuries are related to large team sports such as football, handball and basketball (2-4) preventive actions are warranted. Prevention of acute sport injuries requires four stages: (1) identifying the magnitude of the problem (epidemiology), (2) identifying risk factors that may contribute to injuries (etiology), (3) introducing a preventive measure (prevention), and (4) assessing prevention effectiveness by repeating step 1.(5) To obtain comparable data there are guidelines for standardizations of injury definitions and data collection in the literature.(5-10) In football, rugby union, tennis, cricket and thoroughbred horse racing there are Consensus statements on these procedures.(6, 10-13)

Since most countries do not have a national sports injury surveillance system where injury information from several sports is found in the same database, direct comparisons between sports are difficult to perform (1). Data on sports injury epidemiology may instead be obtained from emergency departments, (2, 14) insurance companies, (15-18) other records for specific sports, (19, 20) or from a specific competition. (8, 21, 22) Studies have found that data from insurance companies could be eligible in sport injury research.(16, 23, 24) Finch (2003) evaluated data from three insurance companies in Australia, compared these data with the desirable and necessary information suggested by ASIDD (The Australian Sports Injury Data Dictionary),(25) and found that relatively high quality sports and recreational injury data can be obtained from insurance claims. (23)

At the time of the present study, 57 out of 70 sport federations under The Swedish Sports Confederation (RF) use the same Swedish insurance company for injury insurance including all organized athletes in each sport federation. This type of insurance covers the athlete for costs associated with their care and treatment after an accidental injury. If the injury results in a medical disability the athlete receives a lump sum in relation to the severity of the disability. In the case of death, a lump sum is paid to the family. All injuries and payments are registered in a database which covers 90% of all athletes in Sweden, at all ages and all levels of sport. Since previous studies have shown that data from insurance companies can be useful in sports injury research, (16, 23, 30) the aim of this study was to evaluate the use of the database from this particular Swedish insurance company in injury preventive research, and to compare it to ASIDD and Consensus statements on sport injuries .

Specific aims:

For peer review only

Specific aims of the study were to measure the reliability of the sports injury data by reviewing the injury data collection procedures and a sample of injuries within the database. The validity of the data was examined by comparing the included variables within the database with variables in ASIDD and Consensus statements from the literature on injury definitions and data collection procedures.(6, 10-13, 23, 25) After these evaluations, improvements to the database will be suggested.

METHODS

A sports injury can be registered with the Swedish insurance company within three years from the time the accident occurred. The definition of an injury eligible for an insurance claim is a new accident injury related to sports activities, training or competition organized by the sports federation or sports club and that is reported to the insurance company. The cause of the injury should be a sudden external force and no overuse injuries are registered. Information regarding the injury and claim is routinely kept in a systematic and structured manner within the sports injury database and is updated over time as new information is available. The majority of the diagnoses are made by doctors. From the database, details can be transformed into an Excel file for statistical analysis. The insurance company performs their own audit of the administrators' claim processing. Randomly selected injury claims, a percentage of the total number of claims, are reviewed by senior administrators every month. Once a year the company auditors also undertake an extensive review of randomly selected injury claims. The latest audit on sports injury claims was performed in the autumn of 2011, and concluded that claim settlements were generally performed with high quality and accuracy.

Randomly selected settled injury claims between 2004 and 2010 including 27 947 unique injuries was exported to an Excel file, and access to a local database for text information surrounding the injury, was received from the insurance company. Data included information on sports activity, age, gender, residence at time of injury, date of injury, diagnosis code (type of injury, injured body region and body part, left or right side of body), and type of financial compensation (medical treatment, dental treatment, permanent disability or death). The data also contained information on disability assessment, the administrator, and costs. The local database contained text files with descriptions of injury mechanisms, training or competition, surface and environment, and protective equipment. The local database also included information on the administrator´s procedure.

For peer review only

To ensure data reliability, the database was reviewed for available and missing information based on desirable and necessary information previously defined in sports injury research. (6, 10-13, 25) From the 27 947 unique injuries, 310 random samples were chosen to evaluate data consistency and lack of information within the Excel file compared to text files in the local database. The insurance company's claim and documentation procedures were studied. All administrators, all sports, and all ages of athletes were represented in the randomly selected samples. Data validity was examined both by comparing the injury data with ASIDD guidelines, in order to make comparisons between different sports (25), and it was also examined relative to Consensus statements in specific sports.(6, 10-13)

The Australian Sports Injury Data Dictionary, ASIDD (1997) provide standardized guidelines for injury data collection .(25) According to ASIDD "*Core"* items (category 1) should be presented in all sports injury data collections, "*Strongly recommended"* items (category 2) should be included, where possible, to give detailed additional injury information and "*Recommended"* items (category 3) can provide further data pertaining to injury circumstances (Table 1). (23) Comparison of the Swedish insurance company data with ASIDD was performed and a scoring system was used to assess the amount of information in the insurance claims according to ASIDD guidelines: (23)

1 Data item fully present (should be fully coded according to ASIDD guidelines)

0 Data item totally absent (in ASIDD but not in the insurance database)

0.5 Data item partially present (some, but not all, of the details specified in ASIDD were in the database)

In order to ensure the validity of the data for specific sports, a comparison with five existing Consensus statements on injury definitions and data collection procedures in the literature (6, 10-13) was performed.

Statistics

The computer programs Microsoft office Excel 2007 and SPSS (SPSS for Windows, version 21.0) were used. Reliability of the claims and documentation procedures are expressed as a percentage by dividing the number of missing values with the number of reviewed posts. When validating the insurance data with ASIDD, a data category agreement score was calculated as the sum of the individual scored items. An overall agreement score calculated as the sum of the three data category agreements to determine the general quality of information. (23) Validation of the data using Consensus statements was based on the established

For peer review only

information criteria published in the scientific literature for football, rugby union, tennis, cricket and thoroughbred horse racing. (6, 10-13)

Ethical approval

The project is a part of a more extensive investigation which has been approved by the Regional Ethical Committee in Stockholm (Dnr 2012/1436-31/1). Data was decoded from identification numbers, and only the research leaders have access to the data. No individual consent of the athletes has been requested as results will be presented on a group level and the insurance policy also informs the policyholders that data can be used in research.

RESULTS

Loss of data

Excel file

Lack of a valid social security number occurred in 103 of 27 947 posts (0,4%) in the Excel file. This was due to foreign athletes practicing their sport in Sweden without a Swedish social security number. The gender and age of these athletes can be determined from the text files in the local database. Fifty-five of 27 947 injuries (0.2%) lacked a diagnosis code but all codes could be identified when reading the texts in the database. Residence at the time of injury (postal code) was missing in approximately 23 % of the posts.

Local database (text files)

Text files in the local database were available for more than 80 % of the 310 random samples taken from the total number of claims. From 2011 onwards, text files were presented in almost all the claims. In some claims from 2007 and earlier, the texts were not always in the local database but could be ordered in hard copy format, which are not included in this study. The diagnosis codes were consistent in more than 88 % of the samples. Incorrect diagnoses were found in three samples (1%), unknown diagnoses in six samples (2%), and correct location but incorrect injury type in 13 samples (4%).

An evaluation of different disciplines within the sport federation was also performed. For example, the Swedish Ski Association has different disciplines within the sport, and in the database there are different category numbers for alpine skiing, cross country skiing, snowboarding etc. The Swedish Mixed Martial Arts Federation and the Swedish Motorsport Federation also have different disciplines within the sport, and thus different category numbers in the data base. All samples tested were found to be in the correct sport, however consistency between the category number in the Excel file and the discipline described in the text file in the local database was approximately 83%.

For peer review only

Compliance to ASIDD

Comparison with ASIDD showed that 93 % (i.e. 28 of 30) of the suggested data items were present, to at least some extent. The overall agreement score was 24 out of a highest possible score of 30 (Table 1). Within "Core" ASIDD items, no missing data were found. All "strongly recommended" items were present in some form but full information was missing in four of them (Table 1). In "recommended" items, five out of eight were present but three of them did not include full information (Table 1). Part of the information regarding ASIDD items was found in the text files within the local database and not in the Excel file.

Table 1. Comparison of the insurance database with ASIDD:

**ASIDD "Core" data item**	**In the insurance data file**
Date of injury	yes: 1
Age	yes: 1
Gender	yes: 1
Activity when injured - broad areas	yes: 1
Mechanism of injury	yes: 1
Body region and body chart	yes: 1
Nature of injury	yes: 1
**Category agreement score** (maximum score = 7)	7 (100%)
**ASIDD "Strongly Recommended" data item**	**In the insurance data file**
Person recording case information	yes: 1
Immediate source of injury record	yes: 1
Area of usual residence	yes: 1
Name of injury place - text	yes: 1
Place of injury - type	yes: 1
Sport and recreation places - specific	yes: 1
Activity when injured -name of sport or activity	yes: 1
Injury factors	yes: 1
Equipment used with intent to protect against injury	yes: 0.5
Narrative of mechanism of injury	yes: 1
Date of presentation	yes: 1
Treatment	yes: 0.5
Advice given to injured person	yes: 0.5
Referral	yes: 0.5
Treating person	yes: 1
**Category agreement score** (maximum score = 15)	13 (87%)
**ASIDD "Recommended" data item**	**In the insurance data file**
Time of injury	no: 0
Date of injury record	yes: 1
Part of specific injury place	yes: 0.5
Phase or aspect of involvement in activity	yes: 0.5
Grade/level of play	yes: 0.5
Specific structure injured	yes: 0.5
Time of presentation	no: 0
Reason for presentation	yes: 1
**Category agreement score** (maximum score = 8)	4 (50%)

Compliance with Consensus statements

Compliance with required information in the Consensus statements, published in sports-related scientific literature, were high (13 of 18 items) except for body mass, height, dominant leg/arm, position of play, and date of returning to sport after injury (Table 2). Some differences in desirable information were found among the different sport federations. Information from seven items existed in the insurance database in some, but not all, claims (Table 2).

Table 2. Comparison between the information from the insurance database with required information in the Consensus statements.

Possible items	Football	Rugby	Tennis	Cricket	Thoroughbred horse racing	Insurance data
Age	x	x	x	x	x	x
Gender	x	x	x	x	x	x
Body mass	x	x	x		x	
Height	x	x	x		x	
Dominant leg/arm	x		x			
Standard/Level of play		x	x		x	x -
Position in play	x	x		x		
Date of onset	x	x	x	x	x	x
Injury diagnosis/Nature of injury	x	x	x	x	x	x
Injured side	x	x	x	x	x	x
Match /training	x	x	x	x	x	x-
Circumstances surrounding the injury	x	x	x	x	x	x -
Date of return to sport	x	x	x		x	
Mechanisms	x	x	x	x	x	x -
Free text (around the injury)	x		x			x -
Protective equipment					x	x -
Surface (playing ground)			x		x	x -
Treatment (medical)				x	x	x -

x - data exist in some but not in all claims

DISCUSSION

As most countries do not have a national sports injury surveillance system where sport injury information from several sports is found in the same database, except for the US where there are national sports injury surveillance systems for adolescents in high school and collegiate athletes. (27, 28) insurance data is a possible way of obtaining this type of information (25). This study confirms that relatively high quality sports injury data can be obtained from a sports injury database such as this specific insurance company in Sweden. The database

For peer review only

included all athletes, in all ages and at different training and competing levels, in 57 sports federations.

In sports injury research, epidemiology and etiology are essential as a foundation for sport injury prevention.(5) It is well known that variations in definitions and methodologies create differences in the results and conclusions from studies of sports injuries. (5, 29, 30) It could therefore be difficult to compare results from different studies. The database in this study includes all licensed and unlicensed athletes in 57 sports federations and the definitions and data collection procedures are the same regardless of sport, thus comparisons between sports, gender and ages are easily made. Since the database only includes accidental injuries and no data of overuse injuries is available, it will not cover the whole injury panorama in each sport.

Earlier studies have shown that insurance claims have the potential to provide detailed information about sports injuries, the records are routinely kept in a systematic manner, and comparisons between data can easily be made.(16, 23, 30) Different studies on sports injuries have used insurance data for different sports, such as netball, rugby and football.(16-18, 24) One smaller study in Finland compared injury profiles in football, ice hockey, volleyball, basketball, judo and karate using national insurance register data.(31) Insurance databases have historically been invaluable for guiding road and workplace safety.(32-34) A recent published study on administrative databases, including insurance databases, concluded that they could be a robust research tool. They allow for longitudinal designs, incidence calculation, and large sample size and power, even for rare events.(30)

The insurance data in the present study included many of the ASIDD items; all “core” and “strongly recommended” items and more than 60% of the “recommended” items were present. (25) Data items that, according to ASIDD, could be improved, related to treatment, referral and advice given to the athlete when injured.(25) Precise and specific information about injury location, phase or aspect of activity, equipment used and specific structure injured was also missing.(25) One detail not registered was the grade or level of play, although it could occasionally be read in the text file that the athlete competed in a national championship.

The results from comparing the injury claim data with desirable information in the Consensus statements (6, 10-13) show that the insurance database could be useful in research on sport

For peer review only

injuries. However, missing data on personal information such as height, body mass, dominant leg/arm, position of play, and return to play are important in some research. Information about return to play is often used to calculate the absence of sporting activity and hence the severity of the injury.(5) In the insurance data, injury type and level of permanent medical disability are two methods of grading injury severity. The definition of medical disability is set by "Insurance Sweden" (the industry organization for insurance companies in Sweden). http://www.svenskforsakring.se/Global/Invaliditetsintyg/Medicinsk_invaliditet_skador120911.pdf

Prospective data collection procedure is considered to be the most appropriate method for sport injury surveillance. (6, 10-13) When using insurance claims data, both retrospectively and prospectively study design, can be used depending on the starting point of the study. In the present study data was retrospectively analyzed.

Van Mechelen suggests that an adequate sports injury surveillance system should be sensitive enough to answer "how many, how often, how long, and serious?".(35) If these questions are to be answered on a national level, where the magnitude of sports injury problems are investigated by comparing incidence rates between various sports, or detecting trends and changes over time, a system that can detect large numbers of cases is needed.(35) The system should be as simple and unambiguous as possible and should at least contain a universally applicable definition of sports injury, of sports participation and consensus on population at risk and time at risk.(35) The insurance data in this study are contained in a national register which covers all athletes in more than 80% of all sport federations, and includes, for example, sports with many athletes such as football, ice hockey, handball, floorball, golf, material arts and motor sports.

Lack of full information within some items both compared with ASIDD and the Consensus statements suggests that the insurance data could be improved upon in the future with the collection of additional details and standardizations of data variables. Some of these changes are implemented in the present insurance database as the result of this study. For example, by using headlines in the database, the terminology regarding injury mechanisms and injury circumstances, match/training, surface and free text around the injury have been standardized based on desirable sport injury information in the literature. (6, 10-13, 25) This, enhance the possibility for future prospective studies of purpose-design surveillance at national level, with a large number of athletes and long follow up time.

For peer review only

Missing desirable information in the insurance data, including total number of athletes in each sport and exposure to sport, could be a weakness when performing epidemiological studies on sport injuries.(5, 36) The number of participants and licensed athletes in each sport could be obtained from the specific sports federations, and exposure to sport might be estimated, also using information from the sports federations.

Despite the quality of the sports injury surveillance system, one will always be confronted with some sort of bias, recall bias, over- or underestimation of sports participation, incomplete responses, non-response, invalid injury description etc.(35) The insurance data collection is structured and routinely kept in a systematic manner, and the insurance company works to keep the data accurate and truthful. The database population consists of athletes who *report* an injury to the insurance company and therefore the data it contains likely underestimates the number of injuries. A study from 2005 on anterior cruciate ligament injuries in football, floorball, handball and ice hockey used the same insurance data as the present study and compared it to two medical databases to determine recording rate reliability.(18) The result showed that 74% of all injuries sustained were recorded in the insurance database.(18) Roos et al (1995) also studying the same database estimated that 98% of the football injuries were registered within seven years.(37) The amount of financial compensation for each claim from the insurance company may influence the rate of injury registration. More severe injuries that require medical treatment or may result in a permanent medical disability are more likely to be reported, as well as dental injuries where treatment is expensive. The athlete can report the injury within three years (sometimes up to ten years) after the accident, and medical disabilities are assessed in one to two years after the accident occurred. This may result in a delay of recording in the database which must be considered when analyzing the data.(37) Despite these weaknesses, the data are important for the detection of serious injuries and injury mechanisms in different sports at national level. Injury data, such as incidence and severity of injury can be compared between sports, gender and ages and be followed over time, for example in longitudinal studies of injury prevention measures at a national level.

CONCLUSION

Before preventative actions can be suggested, sports injury epidemiology, risk factors and injury mechanisms need to be compiled. Since a comprehensive national database from a Swedish insurance company contains most of the desirable information in sport injury research, and further improvements to the database information have been implemented, this

For peer review only - http://bmjopen.bmj.com/site/about/guidelines.xhtml

database now has the potential to be a base for research on acute (accidental) injuries in organized sports at a national level. It could also constitute a foundation for assessment of sport injury prevention at a national level.

Funding

This work was supported by Folksam, a Swedish insurance company

Contributor ship statement

The corresponding author and co-authors have all substantial contributed to the research and to the final document by intellectual content, conception and design, analysis and interpretation of data as well as drafting and critical revision of the manuscript.

Data sharing statement

No additional data available.

Competing Interests

None

For peer review only

REFERENCES

1. Caine D, Caine C, Maffulli N. Incidence and distribution of pediatric sport-related injuries. *Clin J Sport Med.* 2006;**16**:500-13. PubMed PMID: 17119363. Epub 2006/11/23. eng.

2. Conn JM, Annest JL, Gilchrist J. Sports and recreation related injury episodes in the US population, 1997-99. *Inj Prev.* 2003;**9**:117-23. PubMed PMID: 12810736. Pubmed Central PMCID: 1730974.

3. (EuroSafe) EAfIPaSP. Injuries in the European Union. Summary of injury statistics for the years 2008-2010. 2013 (4).

4. Knowles SB, Marshall SW, Bowling JM et al. A prospective study of injury incidence among North Carolina high school athletes. *Am J Epidemiol.* 2006;**164**:1209-21. PubMed PMID: 17012366. Epub 2006/10/03. eng.

5. van Mechelen W, Hlobil H, Kemper HC. Incidence, severity, aetiology and prevention of sports injuries. A review of concepts. *Sports Med.* 1992;**14**:82-99. PubMed PMID: 1509229.

6. Fuller CW, Ekstrand J, Junge A et al. Consensus statement on injury definitions and data collection procedures in studies of football (soccer) injuries. *Clin J Sport Med.* 2006;**16**:97-106. PubMed PMID: 16603877.

7. Finch C. A new framework for research leading to sports injury prevention. *J Sci Med Sport*. 2006;**9**:3-9; discussion 10. PubMed PMID: 16616614. Epub 2006/04/18. eng.

8. Junge A, Engebretsen L, Alonso JM et al. Injury surveillance in multi-sport events: the International Olympic Committee approach. *Br J Sports Med.* 2008;**42**:413-21. PubMed PMID: 18390916. Epub 2008/04/09. eng.

9. Hammond LE, Lilley J, Ribbans WJ. Coding sports injury surveillance data: has version 10 of the Orchard Sports Injury Classification System improved the classification of

For peer review only

sports medicine diagnoses? *Br J Sports Med.* 2009;**43**:498-502. PubMed PMID: 19050000. Epub 2008/12/04. eng.

10. Turner M, Fuller CW, Egan D et al. European consensus on epidemiological studies of injuries in the thoroughbred horse racing industry. Br J Sports Med. 2012;**46**:704-8. PubMed PMID: 22067282. Epub 2011/11/10. eng.

11. Fuller CW, Molloy MG, Bagate C et al. Consensus statement on injury definitions and data collection procedures for studies of injuries in rugby union. *Clin J Sport Med.* 2007;**17**:177-81. PubMed PMID: 17513907. Epub 2007/05/22. eng.

12. Pluim BM, Fuller CW, Batt ME et al. Consensus statement on epidemiological studies of medical conditions in tennis, April 2009. *Br J Sports Med.* 2009;**43**:893-7. PubMed PMID: 19900956. Epub 2009/11/11. eng.

13. Orchard J, Newman D, Stretch R et al. Methods for injury surveillance in international cricket. *J Sci Med Sport.* 2005;**8**:1-14. PubMed PMID: 15887896. Epub 2005/05/13. eng.

14. Nelson NG, McKenzie LB. Mountain biking-related injuries treated in emergency departments in the United States, 1994-2007. *Am J Sports Med.* 2011;**39**:404-9. PubMed PMID: 21076012. Epub 2010/11/16. eng.

15. de Loes M, Dahlstedt LJ, Thomee R. A 7-year study on risks and costs of knee injuries in male and female youth participants in 12 sports. *Scand J Med Sci Sports.* 2000;**10**:90-7. PubMed PMID: 10755279. Epub 2001/02/07. eng.

16. Otago L, Peake J. The role of insurance data in setting priorities for netball injury prevention strategies. *J Sci Med Sport.* 2007;**10**:105-9. PubMed PMID: 16844407.

17. Soderman K, Pietila T, Alfredson H et al. Anterior cruciate ligament injuries in young females playing soccer at senior levels. *Scand J Med Sci Sports.* 2002;**12**:65-8. PubMed PMID: 12121422. Epub 2002/07/18. eng.

For peer review only

18. Forssblad M, Weidenhielm L, Werner S. Knee surgery costs in football, floor ball, European team handball and ice hockey. Scand J Med Sci Sports. 2005 Feb;15(1):43-7. PubMed PMID: 15679571. Epub 2005/02/01. eng.

19. Westin M, Alricsson M, Werner S. Injury profile of competitive alpine skiers: a five-year cohort study. *Knee Surg Sports Traumatol Arthrosc.* 2012;**20**:1175-81. PubMed PMID: 22349602. Epub 2012/02/22. eng.

20. Junge A, Dvorak J. Injury surveillance in the World Football Tournaments 1998-2012. *Br J Sports Med*. 2013;**47**:782-8. PubMed PMID: 23632746. Pubmed Central PMCID: PMC3717759. Epub 2013/05/02. eng.

21. Engebretsen L, Soligard T, Steffen K et al. Sports injuries and illnesses during the London Summer Olympic Games 2012. *Br J Sports Med.* 2013;**47**:407-14. PubMed PMID: 23515712. Epub 2013/03/22. eng.

22. Engebretsen L, Steffen K, Alonso JM et al. Sports injuries and illnesses during the Winter Olympic Games 2010. *Br J Sports Med.* 2010;**44**:772-80. PubMed PMID: 20820057. Epub 2010/09/08. eng.

23. Finch C. How useful are insurance claim data for sports injury prevention purposes? *Inj Control Saf Promot.* 2003 Sep:181-3.

24. King DA, Hume PA, Milburn P et al. Rugby league injuries in New Zealand: a review of 8 years of Accident Compensation Corporation injury entitlement claims and costs. *Br J Sports Med.* 2009;**43**:595-602. PubMed PMID: 19553223. Epub 2009/06/26. eng.

25. Party TASIDW. Australian Sports Injury Data Dictionary. Guidelines for injury data collection and classification for the prevention and control of injury in sport and recreation. Working dokument. 1997.

26. Kucera K, Marshall S, Bell D et al. Validity of soccer injury data from the national collegiate athletic assosiations injury surveillance system. *J of Athl.Train.* 2011;**46**:489-499.

For peer review only

27. Robinson W, Corlette J, Collins C et al. Shoulder injuries among US high school athlete, 2005/2006 - 2011/2012. *Pediatrics* 2014;**133**: 272-279.

28. Brooks J, Fuller C. The influence of methodological issues on the results and conclusions from epidemiological studies of sports injuries. Illustrative examples. *Sports Med.* 2006;**36**:459-472.

29. Finch C. An overview of some definitional issues for sports injury surveillance. *Sports Med.* 1997;**24**:157-163.

30. Gavrielov-Yusim N, Friger M. Use of administrative medical databases in population-based research. J Epidemiol Community Health. 2013 Nov 18. PubMed PMID: 24248997. Epub 2013/11/20. Eng.

31. Kujala UM, Taimela S, Antti-Poika I et al. Acute injuries in soccer, ice hockey, volleyball, basketball, judo, and karate: analysis of national registry data. *BMJ.* 1995;**311**:1465-8. PubMed PMID: 8520333. Pubmed Central PMCID: PMC2543722. Epub 1995/12/02. eng.

32. Badii M, Keen D, Yu S et al. Evaluation of a comprehensive integrated workplace-based program to reduce occupational musculoskeletal injury and its associated morbidity in a large hospital. *Journal of occupational and environmental medicine / American College of Occupational and Environmental Medicine.* 2006;**48**:1159-65. PubMed PMID: 17099452. Epub 2006/11/14. eng.

33. McCall BP, Horwitz IB, Taylor OA. Occupational eye injury and risk reduction: Kentucky workers' compensation claim analysis 1994-2003. *Inj Prev.* 2009;**15**:176-82. PubMed PMID: 19494097. Epub 2009/06/06. eng.

34. Krafft M, Kullgren A, Lie A et al. The use of seat belts in cars with smart seat belt reminders--results of an observational study. *Traffic Inj Prev.* 2006;**7:**125-9. PubMed PMID: 16854706. Epub 2006/07/21. eng.

For peer review only

35. van Mechelen W. Sports injury surveillance systems. 'One size fits all'? *Sports Med.* 1 997;**24**:164-8. PubMed PMID: 9327530.

36. de Loes M. Exposure data. Why are they needed? *Sports Med.* 1997;**24**:172-5. PubMed PMID: 9327532. Epub 1997/11/05. eng.

37. Roos H, Ornell M, Gardsell P et al. Soccer after anterior cruciate ligament injury--an incompatible combination? A national survey of incidence and risk factors and a 7-year follow-up of 310 players. *Acta orthopaedica Scandinavica.* 1995;**66**:107-12. PubMed PMID: 7740937. Epub 1995/04/01. eng.

For peer review only

Title

Insurance claims data - a possible solution for a national sports injury surveillance system? at a national level; evaluation of insurance claims data

Corresponding author
Malin Åman
GIH -The Swedish School of Sport and Health and Sciences
Lidingövägen 1, Box 5626, 114 86 Stockholm, Sweden.
malin.aman@gih.se
telephone +46739947349, fax +46812053880

Co-authors
Magnus Forssblad
Department of Molecular Medicine and Surgery, Karolinska Institut,
Stockholm Sports Trauma Research Center, Capio Artro Clinic,
Box 5605, 114 86 Stockholm, Sweden

Karin Henriksson-Larsén
GIH -The Swedish School of Sport and Health and Sciences
Lidingövägen 1, Box 5626, 114 86 Stockholm, Sweden.

Keywords
Sports injury, athletic injury, accident prevention, recreation, epidemiology

Word count

3 1722 792

Number of references

37

33

For peer review only

ABSTRACT

Background:

Before preventive actions can be suggested for sports injuries at a national level a solid surveillance system is required in order to study~~,~~ epidemiology, risk factors and mechanisms. There ~~need to be inventoried and there~~ are guidelines for sport injury data collection and classifications in the literature for that purpose.~~.~~ In Sweden, 90% of all athletes, (~~and~~ 57/70 sport federations)~~federation~~ are insured in the same insurance company and the data from their database could be a foundation for studies on acute sports injuries, at a national level.

Formatted: Font color: Red

Formatted: Font color: Red

Objective:

To evaluate the usefulness of sports injury insurance claims in sports injury surveillance, at a national level.

Method:

A database ~~An Excel file~~ with 27947 injuries was exported to an excel file. Access~~, as well as access~~ to the corresponding~~database for~~ text files~~information~~ was also obtained~~used.~~ Data were reviewed on available information, missing information, and drop-outs. Comparison against ASIDD (Australian Sports Injury Data Dictionary) and with existing Consensus statement in the literature (Football (soccer), Rugby union, Tennis, Cricket and Thoroughbred horse racing) was performed in a structured manner.

Result:

Comparison with ASIDD showed that 93 % of the suggested data items were present in the database, to at least some extent. Compliance with the Consensus statements was generally high (13/18). Almost all claims (83%) contained text information concerning~~surrounding~~ the injury.

Conclusion:

Relatively high quality sports injury data can be obtained from a specific insurance company at national level in Sweden. The database has the potential to be a solid base for research on acute sport injuries in different sports~~at a national level~~.

For peer review only

Strengths and limitations of this study:

• DataIt includes a large material from a database that coverscowers more than 80% of the sports federations in Sweden at a national level.

• The studyIt is a structured examination of the reliability and validity of data, using standardized guidelines from the sport injury literature.

• A limitation is that data is not evaluated for recording rate reliability since there is no national record of sports injuries in the country.

For peer review only

INTRODUCTION

Since approximately 18-30 % of all acute injuries are sports-related (1, 2), and almost half these injuries are related to large team sports such as football, handball and basketball (2-4)accidental injuries) are sports-related, preventive actions are warranted. It is suggested that almost half these injuries are related to team sports, in particular soccer, handball and basketbalThis type of injury, a traumatic injury, is defined as an injury resulting from a specific, identifiable ePrevention of acute sport injuries requires four stages: (1) identifying the magnitude of the problem (epidemiology), (2) identifying risk factors that may contribute to injuries (etiology), (3) introducing a preventive measure (prevention), and (4) assessing prevention effectiveness by repeating step 1.(5) To obtain comparable data thereThere are guidelines for standardizations of injury definitions and data collection in the literature.(5-10) In football, rugby union, tennis, cricket and thoroughbred horse racing there are Consensus statements on these procedures.(6, 10-13) In order to perform comparative studies it is important to have a standardized method that allows for comparison between various types of sports.

Since mostMost countries do not have a national sports injury surveillance system where injury information from several sports is found in the same database, direct comparisons between sports are difficult to perform (1).. Data onfor sports injury epidemiology may instead be obtained from emergency departments, ,(2, 14) insurance companies, (15-18) other records for specific sports, (19, 20) or from a specific competition. (8, 21, 22) Studies have found that data from insurance companies could be eligible in sport injury research.(16, 23, 24) Finch (2003) evaluated data from three insurance companies in Australia, compared these data with the desirable and necessary information suggested by ASIDD (The Australian Sports Injury Data Dictionary),(25) and found that relatively high quality sports and recreational injury data can be obtained from insurance claims. (23)

For peer review only

At the time of the present study, 57 out of 70 sport federations under The Swedish Sports Confederation (RF) use the same Swedish insurance company for injury insurance including all organized athletes in each sport federation.~~-~~ This type of insurance covers the athlete for costs associated with their care and treatment after an accidental injury. If the injury results in a medical disability the athlete receives a lump sum in relation to the severity of the disability. In the case of death, a lump sum is paid to the family. All injuries and payments are registered in a database which covers 90% of all athletes in Sweden, at all ages and all levels of sport.~~-~~ Since previous studies have shown that data from insurance companies can be useful in sports injury research, (16, 23, 30~~26~~) the aim of this study was to evaluate the use of the database from this particular Swedish insurance company in injury preventive research, and to compare it to ASIDD and Consensus statements on sport injuries .

Specific aims:

Specific aims of the study were to measure the reliability of the sports injury data by reviewing the injury data collection procedures and a sample of injuries within the database. The validity of the data was examined by comparing the included variables within the database with variables in ASIDD and Consensus statements from the literature on injury definitions and data collection procedures.(6, 10-13, 23, 25) After these evaluations, improvements to the database will be suggested.

METHODS

A sports injury can be registered with the Swedish insurance company within three years from the time the accident occurred. The definition of an injury eligible for an insurance claim is a new accident injury related to sports activities, training or competition organized by the sports federation or sports club and that is reported to the insurance company. The cause of the injury should be a sudden external force and no overuse injuries are registered~~receiving medical attention~~. Information regarding the injury and claim is routinely kept in a systematic and structured manner within the sports injury database and is updated over time as new information is available. The majority of the diagnoses are made by doctors.~~-~~ From the database, details can be transformed into an Excel file for statistical analysis. The insurance company performs their own audit of the administrators' claim processing. Randomly selected injury claims, a percentage of the total number of claims, are reviewed by senior administrators every month. Once a year the company auditors also undertake an extensive review of randomly selected injury claims. The latest audit on sports injury claims was

For peer review only

performed in the autumn of 2011, and concluded that claim settlements were generally performed with high quality and accuracy.

R~~r~~andomly selected~~An Excel file containing 35 511 posts of~~ settled injury claims between 2004 and 2010 including 27 947 unique injuries was exported to an Excel file~~,~~ and access to a local database for text information surrounding the injury, was received from the insurance company. Data ~~in the Excel file~~ included information on sports activity, age, gender, residence at time of injury, date of injury, diagnosis code (type of injury, injured body region and body part, left or right side of body), and type of financial compensation (medical treatment, dental treatment, permanent disability or death). The data~~file~~ also contained information on disability assessment, the administrator, and costs. The local database contained text files with descriptions of injury mechanisms, training or competition, surface and environment, and protective equipment. The local database also included information on the administrator´s procedure.

To ensure data reliability, the database~~Excel file~~ was reviewed for available and~~information,~~ missing information based on desirable and necessary information previously defined in sports injury research. (6, 10-13, 25) From the 27 947 unique injuries~~Excel file~~, 310 random samples were chosen to evaluate data consistency and lack of information within the Excel file compared to text files in the local database. The insurance company's claim and documentation procedures were studied. All administrators, all sports, and all ages of athletes were represented in the randomly selected samples. Data validity was examined both by comparing the injury data with ASIDD guidelines, in order to make comparisons between different sports (25), and it was also examined relative to Consensus statements in specific sports.(6, 10-13)

The Australian Sports Injury Data Dictionary, ASIDD (1997) ~~was developed in Australia to~~ provide standardized guidelines for injury data collection ~~and classification in order to help prevent and control injury in sport and recreation. .~~(25) According to ASIDD ~~states that~~ "*Core"* items (category 1) should be presented in all sports injury data collections, "*Strongly recommended"* items (category 2) should be included, where possible, to give detailed additional injury information and "*Recommended"* items (category 3) can provide further data pertaining to injury circumstances (Table 1). (23) Comparison of the Swedish insurance

For peer review only

company data with ASIDD was performed and a scoring system was used to assess the amount of information in the insurance claims according to ASIDD guidelines: (23)

1 Data item fully present (should be fully coded according to ASIDD guidelines)

0 Data item totally absent (in ASIDD but not in the insurance database)

0.5 Data item partially present (some, but not all, of the details specified in ASIDD were in the database)

In order to ensure the validity of the data for specific sports, a comparison with five existing Consensus statements on injury definitions and data collection procedures in the literature (6, 10-13) was performed.

Statistics

The computer programs Microsoft office Excel 2007 and SPSS (SPSS for Windows, version 21.0) were used. Reliability of the claims and documentation procedures are expressed as a percentage by dividing the number of missing values with the number of reviewed posts. When validating the insurance data with ASIDD, a data category agreement score was calculated as the sum of the individual scored items. An overall agreement score calculated as the sum of the three data category agreements to determine the general quality of information. (23) Validation of the data using Consensus statements was based on the established information criteria published in the scientific literature for football, rugby union, tennis, cricket and thoroughbred horse racing. (6, 10-13)

Ethical approval

Formatted: Font: Bold

The project is a part of a more extensive investigation which has been approved by the Regional Ethical Committee in Stockholm (Dnr 2012/1436-31/1). Data was decoded from identification numbers, and only the research leaders have access to the data. No individual consent of the athletes has been requested as results will be presented on a group level and the insurance policy also informs the policyholders that data can be used in research.

RESULTS

Loss of data

Excel file

Lack of a valid social security number occurred in 103 of 27 947 posts (0,4%) in the Excel file. This was due to foreign athletes practicing their sport in Sweden without a Swedish social security number. The gender and age of these athletes can be determined from the text files in the local database. Fifty-five of 27 947 injuries (0.2%) lacked a diagnosis code but all

For peer review only

codes could be identified when reading the texts in the database. Residence at the time of injury (postal code) was missing in approximately 23 % of the posts.

Local database (text files)

Text files in the local database were available for more than 80 % of the 310 random samples taken from the total number of claims. From 2011 onwards, text files were presented in almost all the claims. In some claims from 2007 and earlier, the texts were not always in the local database but could be ordered in hard copy format, which are not included in this study. The diagnosis codes were consistent in more than 88 % of the samples. Incorrect diagnoses were found in three samples (1%), unknown diagnoses in six samples (2%), and correct location but incorrect injury type in 13 samples (4%).

An evaluation of different disciplines~~categories contained~~ within the sport federation~~different sports~~ was also performed. For example, the Swedish Ski Association has different disciplines within the sport, and in the database there are different category numbers~~categories~~ for alpine skiing, cross country skiing, snowboarding etc. The Swedish Mixed Martial Arts Federation and the Swedish Motorsport Federation also have different disciplines within the sport, and thus different category numbers in the data base.~~categories.~~ All samples tested were found to be in the correct sport, however consistency between the category number in the Excel file and the discipline described in the text file in the local database was approximately 83%.

Compliance to ASIDD

Comparison with ASIDD showed that 93 % (i.e. 28 of 30) of the suggested data items were present, to at least some extent. The overall agreement score was 24 out of a highest possible score of 30 (Table 1). Within "Core" ASIDD items, no missing data were found. All "strongly recommended" items were present in some form but full information was missing in four of them (Table 1). In "recommended" items, five out of eight were present but three of them did not include full information (Table 1). Part of the information regarding ASIDD items was found in the text files within the local database and not in the Excel file.

Table 1. Comparison of the insurance database with ASIDD:

ASIDD "Core" data item	In the insurance data file
Date of injury	yes: 1
Age	yes: 1
Gender	yes: 1
Activity when injured - broad areas	yes: 1
Mechanism of injury	yes: 1
Body region and body chart	yes: 1
Nature of injury	yes: 1

For peer review only

Category agreement score (maximum score = 7)	7 (100%)
**ASIDD "Strongly Recommended" data item**	**In the insurance data file**
Person recording case information	yes: 1
Immediate source of injury record	yes: 1
Area of usual residence	yes: 1
Name of injury place - text	yes: 1
Place of injury - type	yes: 1
Sport and recreation places - specific	yes: 1
Activity when injured -name of sport or activity	yes: 1
Injury factors	yes: 1
Equipment used with intent to protect against injury	yes: 0.5
Narrative of mechanism of injury	yes: 1
Date of presentation	yes: 1
Treatment	yes: 0.5
Advice given to injured person	yes: 0.5
Referral	yes: 0.5
Treating person	yes: 1
**Category agreement score** (maximum score = 15)	13 (87%)
**ASIDD "Recommended" data item**	**In the insurance data file**
Time of injury	no: 0
Date of injury record	yes: 1
Part of specific injury place	yes: 0.5
Phase or aspect of involvement in activity	yes: 0.5
Grade/level of play	yes: 0.5
Specific structure injured	yes: 0.5
Time of presentation	no: 0
Reason for presentation	yes: 1
**Category agreement score** (maximum score = 8)	4 (50%)

Formatted: Centered

Formatted: Centered

Formatted: Centered

Compliance with Consensus statements

Compliance with required information in the Consensus statements, published in sports-related scientific literature, were high (13 of 18 items) except for body mass, height, dominant leg/arm, position of play, and date of returning to sport after injury (Table 2). Some differences in desirable information were found among the different sport federations. Information from seven items existed in the insurance database in some, but not all, claims (Table 2).

Table 2. Comparison between the information from the insurance database with required information in the Consensus statements.

Possible items	Football	Rugby	Tennis	Cricket	Thoroughbred horse racing	Insurance data
Age	x	x	x	x	x	x
Gender	x	x	x	x	x	x
Body mass	x	x	x		x	
Height	x	x	x		x	
Dominant leg/arm	x		x			

For peer review only

Standard/Level of play		x	x		x	x -
Position in play	x	x		x		
Date of onset	x	x	x	x	x	x
Injury diagnosis/Nature of injury	x	x	x	x	x	x
Injured side	x	x	x	x	x	x
Match /training	x	x	x	x	x	x-
Circumstances surrounding the injury	x	x	x	x	x	x -
Date of return to sport	x	x	x		x	
Mechanisms	x	x	x	x	x	x -
Free text (around the injury)	x		x			x -
Protective equipment					x	x -
Surface (playing ground)			x		x	x -
Treatment (medical)				x	x	x -

x - data exist in some but not in all claims

DISCUSSION

As most countries do not have a national sports injury surveillance system where sport injury information from several sports is found in the same database, except for the US where there are national sports injury surveillance systems for adolescents in high school and collegiate athletes. (27, 28) insurance data is a possible way of obtaining this type of information (25). This study confirms~~suggests~~ that relatively high quality sports injury data can be obtained from a sports injury database such as this specific insurance company in Sweden. The database included all athletes, in all ages and at different training and competing levels, in 57 sports federations. ~~, when ASIDD is used as the gold standard. Also, on comparing the data with Consensus statements from studies of sport injuries, the data from the insurance company is shown to be of relatively high quality.~~

In sports injury research, epidemiology and etiology are essential as a foundation for sport injury prevention. (5) It is well known that variations in definitions and methodologies create differences in the results and conclusions from studies of sports injuries. (5, 29, 30) It could therefore be difficult to compare results from different studies. The database in this study includes all licensed and unlicensed athletes in 57 sports federations and the definitions and data collection procedures are the same regardless of sport, thus comparisons between sports, gender and ages are easily made. Since the database only includes accidental injuries and no data of overuse injuries is available, it will not cover the whole injury panorama in each sport.

Formatted: Font color: Red
Formatted: Font color: Red
Formatted: Font color: Red
Formatted: Font color: Red
Formatted: Font color: Red
Formatted: Font color: Red
Formatted: Font color: Red

For peer review only

Earlier studies have shown that insurance claims have the potential to provide detailed information about sports injuries, the records are routinely kept in a systematic manner, and comparisons between data can easily be made.(16, 23, 30~~26~~) Different studies on sports injuries have used insurance data for different sports, such as netball, rugby and football.(16-18, 24) One smaller study in Finland compared injury profiles in football, ice hockey, volleyball, basketball, judo and karate using national insurance register data.(31~~27~~) Insurance databases have historically been invaluable for guiding road and workplace safety.(32-34)~~(28-30)~~ A recent published study on administrative databases, including insurance databases, concluded that they could be a robust research tool. They allow for longitudinal designs, incidence calculation, and large sample size and power, even for rare events.(30)~~(26)~~

The insurance data in ~~this~~ the present study included many of the ASIDD items; all "core" and "strongly recommended" items and more than 60% of the "recommended" items were present. (25) Data items that, according to ASIDD, could be improved, related to treatment, referral and advice given to the athlete when injured.(25) Precise and specific information about injury location, phase or aspect of activity, equipment used and specific structure injured was also missing.(25) One detail not registered was the grade or level of play, although it could occasionally be read in the text file that the athlete competed in a national championship.

The results from comparing the injury claim data with desirable information in the Consensus statements (6, 10-13) show that the insurance database could be useful in research on sport injuries. However, missing data on personal information such as height, body mass, dominant leg/arm, position of play, and return to play are important in some research. Information about return to play is often used to calculate the absence of sporting activity and hence the severity of the injury.(5) In the insurance data, injury type and level of permanent medical disability are two methods of grading injury severity. The definition of medical disability is set by "Insurance Sweden" (the industry organization for insurance companies in Sweden). http://www.svenskforsakring.se/Global/Invaliditetsintyg/Medicinsk_invaliditet_skador120911.pdf

Prospective data collection procedure is considered to be the most appropriate method for sport injury surveillance. (6, 10-13) When using insurance claims data, both retrospectively and prospectively study design, can be used depending on the starting point of the study. In the present study data was retrospectively analyzed.

Van Mechelen suggests that an adequate sports injury surveillance system should be sensitive enough to answer “how many, how often, how long, and serious?”.(35~~31~~) If these questions are to be answered on a national level, where the magnitude of sports injury problems are investigated by comparing incidence rates between various sports, or detecting trends and changes over time, a system that can detect large numbers of cases is needed.(35)~~(31)~~ The system should be as simple and unambiguous as possible and should at least contain a universally applicable definition of sports injury, of sports participation and consensus on population at risk and time at risk.(35~~31~~) The insurance data in this study are contained in a national register which covers all athletes in more than 80% of all sport federations~~sports~~, and includes, for example, sports with many athletes such as football, ice hockey, handball, floorball, golf, material arts and motor sports. ~~Athletes participating in some of these sports are known to be susceptible to many acute injuries.(2, 3)~~

Lack of full information within some items both compared with ASIDD and the Consensus statements suggests that the insurance data could be improved upon in the future with the collection of additional details and standardizations of data variables. Some of these changes are implemented in the present insurance database as the result of this study. For example, by using headlines in the database, the terminology regarding injury mechanisms and injury circumstances, match/training, surface and free text around the injury have been standardized based on desirable sport injury information in the literature. (6, 10-13, 25) This, enhance the possibility for future prospective studies of purpose-design surveillance at national level, with a large number of athletes and long follow up time.

Missing desirable information in the insurance data, including total number of athletes in each sport and exposure to sport, could be a weakness when performing epidemiological studies on sport injuries.(5, 36~~32~~) The number of participants and licensed athletes in each sport could be obtained from the specific sports federations, and exposure to sport might be estimated, also using information from the sports federations.

Despite the quality of the sports injury surveillance system, one will always be confronted with some sort of bias, recall bias, over- or underestimation of sports participation, incomplete responses, non-response, invalid injury description etc.(35~~31~~) The insurance data collection is structured and routinely kept in a systematic manner, and the insurance company works to keep the data accurate and truthful. The database population consists of athletes who *report* an injury to the insurance company and therefore the data it contains likely underestimates the

For peer review only

number of injuries. A study from 2005 on anterior cruciate ligament injuries in football, floorball, handball and ice hockey used the same insurance data as the present study and compared it to two medical databases to determine recording rate reliability.(18) The result showed that 74% of all injuries sustained were recorded in the insurance database.(18) Roos et al (1995) also studying the same database estimated that 98% of the football injuries were registered within seven years.(37~~33~~) The amount of financial compensation for each claim from the insurance company may influence the rate of injury registration. More severe injuries that require medical treatment or may result in a permanent medical disability are more likely to be reported, as well as dental injuries where treatment is expensive. The athlete can report the injury within three years (sometimes up to ten years) ~~of~~ after the accident, and medical disabilities are assessed in one to two years after the accident occurred. This may result in a delay of recording in the database ~~and~~ which must be considered when analyzing the data.(37~~33~~)

Despite these weaknesses, the data are important for the detection of serious injuries and injury mechanisms in different sports at national level. Injury~~The~~ data, such as incidence and severity of injury can ~~also~~ be compared between sports, gender and ages and be followed ~~up~~ over time, for example~~used~~ in longitudinal studies of~~on~~ injury prevention measures at a national level.

CONCLUSION

Before preventative actions can be suggested, sports injury epidemiology, risk factors and injury mechanisms need to be compiled. Since a comprehensive national database~~.(5) In most countries, there is a lack of national sports injury surveillance systems and comparisons of injuries between sports are difficult to perform. Since national data~~ from a Swedish insurance company contains most of the desirable information in sport injury research, and further improvements to the database information have been implemented, this database now has the potential to be a base for research on acute (accidental) injuries in organized~~different~~ sports at a national level. It could also constitute a foundation for assessment of sport injury prevention at a national level.

Formatted: Font color: Red

Formatted: Font color: Red

Formatted: Font color: Red

Formatted: Font color: Red

Formatted: Font: Bold, Font color: Black

For peer review only

Funding

This work was supported by ~~the~~ Folksam, a Swedish insurance company.

Formatted: Font: Times New Roman, 12 pt

Formatted: pagecontents, Font: (Default) Calibri, 11 pt, Font color: Auto

Contributor ship statement

The corresponding author and co-authors have all substantial contributed to the research and to the final document by intellectual content, conception and design, analysis and interpretation of data as well as drafting and critical revision of the manuscript.

Data sharing statement

No additional data available.

Competing Interests

None

REFERENCES

For peer review only

1. Caine D, Caine C, Maffulli N. Incidence and distribution of pediatric sport-related injuries. *Clin J Sport Med.* 2006;**16**:500-13. PubMed PMID: 17119363. Epub 2006/11/23. eng.

Formatted: Line spacing: 1.5 lines

2. Conn JM, Annest JL, Gilchrist J. Sports and recreation related injury episodes in the US population, 1997-99. *Inj Prev.* 2003;**9**:117-23. PubMed PMID: 12810736. Pubmed Central PMCID: 1730974.

3. (EuroSafe) EAfIPaSP. Injuries in the European Union. Summary of injury statistics forstatisticsfor the years 2008-2010. 2013 (4).

4. Knowles SB, Marshall SW, Bowling JM et al. A prospective study of injury incidence among North Carolina high school athletes. *Am J Epidemiol.* 2006;**164**:1209-21. PubMed PMID: 17012366. Epub 2006/10/03. eng.

5. van Mechelen W, Hlobil H, Kemper HC. Incidence, severity, aetiology and prevention of sports injuries. A review of concepts. *Sports Med.* 1992;**14**:82-99. PubMed PMID: 1509229.

6. Fuller CW, Ekstrand J, Junge A et al. Consensus statement on injury definitions and data collection procedures in studies of football (soccer) injuries. *Clin J Sport Med.* 2006;**16**:97-106. PubMed PMID: 16603877.

7. Finch C. A new framework for research leading to sports injury prevention. *J Sci Med Sport*. 2006;**9**:3-9; discussion 10. PubMed PMID: 16616614. Epub 2006/04/18. eng.

8. Junge A, Engebretsen L, Alonso JM et al. Injury surveillance in multi-sport events: the International Olympic Committee approach. *Br J Sports Med.* 2008;**42**:413-21. PubMed PMID: 18390916. Epub 2008/04/09. eng.

Formatted: Swedish (Sweden)

9. Hammond LE, Lilley J, Ribbans WJ. Coding sports injury surveillance data: has version 10 of the Orchard Sports Injury Classification System improved the classification of sports medicine diagnoses? *Br J Sports Med.* 2009;**43**:498-502. PubMed PMID: 19050000. Epub 2008/12/04. eng.

For peer review only

10. Turner M, Fuller CW, Egan D et al. European consensus on epidemiological studies of injuries in the thoroughbred horse racing industry. Br J Sports Med. 2012;**46**:704-8. PubMed PMID: 22067282. Epub 2011/11/10. eng.

11. Fuller CW, Molloy MG, Bagate C et al. Consensus statement on injury definitions and data collection procedures for studies of injuries in rugby union. *Clin J Sport Med.* 2007;**17**:177-81. PubMed PMID: 17513907. Epub 2007/05/22. eng.

12. Pluim BM, Fuller CW, Batt ME et al. Consensus statement on epidemiological studies of medical conditions in tennis, April 2009. *Br J Sports Med.* 2009;**43**:893-7. PubMed PMID: 19900956. Epub 2009/11/11. eng.

13. Orchard J, Newman D, Stretch R et al. Methods for injury surveillance in international cricket. *J Sci Med Sport.* 2005;**8**:1-14. PubMed PMID: 15887896. Epub 2005/05/13. eng.

14. Nelson NG, McKenzie LB. Mountain biking-related injuries treated in emergency departments in the United States, 1994-2007. *Am J Sports Med.* 2011;**39**:404-9. PubMed PMID: 21076012. Epub 2010/11/16. eng.

15. de Loes M, Dahlstedt LJ, Thomee R. A 7-year study on risks and costs of knee injuries in male and female youth participants in 12 sports. *Scand J Med Sci Sports.* 2000;**10**:90-7. PubMed PMID: 10755279. Epub 2001/02/07. eng.

16. Otago L, Peake J. The role of insurance data in setting priorities for netball injury prevention strategies. *J Sci Med Sport.* 2007;**10**:105-9. PubMed PMID: 16844407.

17. Soderman K, Pietila T, Alfredson H et al. Anterior cruciate ligament injuries in young females playing soccer at senior levels. *Scand J Med Sci Sports.* 2002;**12**:65-8. PubMed PMID: 12121422. Epub 2002/07/18. eng.

18. Forssblad M, Weidenhielm L, Werner S. Knee surgery costs in football, floor ball, European team handball and ice hockey. Scand J Med Sci Sports. 2005 Feb;15(1):43-7. PubMed PMID: 15679571. Epub 2005/02/01. eng.

For peer review only

19. Westin M, Alricsson M, Werner S. Injury profile of competitive alpine skiers: a five-year cohort study. *Knee Surg Sports Traumatol Arthrosc.* 2012;**20**:1175-81. PubMed PMID: 22349602. Epub 2012/02/22. eng.

20. Junge A, Dvorak J. Injury surveillance in the World Football Tournaments 1998-2012. *Br J Sports Med*. 2013;**47**:782-8. PubMed PMID: 23632746. Pubmed Central PMCID: PMC3717759. Epub 2013/05/02. eng.

21. Engebretsen L, Soligard T, Steffen K et al. Sports injuries and illnesses during the London Summer Olympic Games 2012. *Br J Sports Med.* 2013;**47**:407-14. PubMed PMID: 23515712. Epub 2013/03/22. eng.

22. Engebretsen L, Steffen K, Alonso JM et al. Sports injuries and illnesses during the Winter Olympic Games 2010. *Br J Sports Med.* 2010;**44**:772-80. PubMed PMID: 20820057. Epub 2010/09/08. eng.

23. Finch C. How useful are insurance claim data for sports injury prevention purposes? *Inj Control Saf Promot.* 2003 Sep:181-3.

24. King DA, Hume PA, Milburn P et al. Rugby league injuries in New Zealand: a review of 8 years of Accident Compensation Corporation injury entitlement claims and costs. *Br J Sports Med.* 2009;**43**:595-602. PubMed PMID: 19553223. Epub 2009/06/26. eng.

25. Party TASIDW. Australian Sports Injury Data Dictionary. Guidelines for injury data collection and classification for the prevention and control of injury in sport and recreation. Working dokument. 1997.

26. Kucera K, Marshall S, Bell D et al. Validity of soccer injury data from the national collegiate athletic assosiations injury surveillance system. *J of Athl.Train.* 2011;**46**:489-499.

27. Robinson W, Corlette J, Collins C et al. Shoulder injuries among US high school athlete, 2005/2006 - 2011/2012. *Pediatrics* 2014;**133**: 272-279.

For peer review only

28. Brooks J, Fuller C. The influence of methodological issues on the results and conclusions from epidemiological studies of sports injuries. Illustrative examples. *Sports Med.* 2006;**36**:459-472.

29. Finch C. An overview of some definitional issues for sports injury surveillance. *Sports Med.* 1997;**24**:157-163.

3026. Gavrielov-Yusim N, Friger M. Use of administrative medical databases in population-based research. J Epidemiol Community Health. 2013 Nov 18. PubMed PMID: 24248997. Epub 2013/11/20. Eng.

3127. Kujala UM, Taimela S, Antti-Poika I et al. Acute injuries in soccer, ice hockey, volleyball, basketball, judo, and karate: analysis of national registry data. *BMJ.* 1995;**311**:1465-8. PubMed PMID: 8520333. Pubmed Central PMCID: PMC2543722. Epub 1995/12/02. eng.

3228. Badii M, Keen D, Yu S et al. Evaluation of a comprehensive integrated workplace-based program to reduce occupational musculoskeletal injury and its associated morbidity in a large hospital. *Journal of occupational and environmental medicine / American College of Occupational and Environmental Medicine.* 2006;**48**:1159-65. PubMed PMID: 17099452. Epub 2006/11/14. eng.

3329. McCall BP, Horwitz IB, Taylor OA. Occupational eye injury and risk reduction: Kentucky workers' compensation claim analysis 1994-2003. *Inj Prev.* 2009;**15**:176-82. PubMed PMID: 19494097. Epub 2009/06/06. eng.

3430. Krafft M, Kullgren A, Lie A et al. The use of seat belts in cars with smart seat belt reminders--results of an observational study. *Traffic Inj Prev.* 2006;**7**:125-9. PubMed PMID: 16854706. Epub 2006/07/21. eng.

3531. van Mechelen W. Sports injury surveillance systems. 'One size fits all'? *Sports Med.* 1997;**24**:164-8. PubMed PMID: 9327530.

Formatted: Line spacing: 1.5 lines

Formatted: Check spelling and grammar

For peer review only

3632. PubMed de Loes M. Exposure data. Why are they needed? *Sports Med.* 1997;**24**:172-5. PMID: 9327532. Epub 1997/11/05. eng.

3733. Roos H, Ornell M, Gardsell P et al. Soccer after anterior cruciate ligament injury--an incompatible combination? A national survey of incidence and risk factors and a 7-year follow-up of 310 players. *Acta orthopaedica Scandinavica.* 1995;**66**:107-12. PubMed PMID: 7740937. Epub 1995/04/01. eng.

For peer review only

BMJ Open

Insurance claims data - a possible solution for a national sports injury surveillance system? – An evaluation of data information against ASIDD and Consensus statements on sports injury surveillance.

Journal:	*BMJ Open*
Manuscript ID:	bmjopen-2014-005056.R2
Article Type:	Research
Date Submitted by the Author:	08-May-2014
Complete List of Authors:	Åman, Malin; The Swedish School of Sport and Health Scienses, Performance and training<br>Forssblad, Magnus; Department of Molecular Medicine and Surgery, Karolinska Institut, Stockholm Sports Trauma Research Center, Capio Artro Clinic<br>Henriksson-Larsén, Karin; The Swedish School of Sport and Health Scienses, Principal
**Primary Subject Heading**:	Sports and exercise medicine
Secondary Subject Heading:	Sports and exercise medicine
Keywords:	SPORTS MEDICINE, EPIDEMIOLOGY, PREVENTIVE MEDICINE, PUBLIC HEALTH

SCHOLARONE™
Manuscripts

Title

Insurance claims data - a possible solution for a national sports injury surveillance system? – An evaluation of data information against ASIDD and Consensus statements on sports injury surveillance.

Corresponding author

Malin Åman
GIH -The Swedish School of Sport and Health and Sciences
Lidingövägen 1, Box 5626, 114 86 Stockholm, Sweden.
malin.aman@gih.se
telephone +46739947349, fax +46812053880

Co-authors

Magnus Forssblad
Department of Molecular Medicine and Surgery, Karolinska Institut,
Stockholm Sports Trauma Research Center, Capio Artro Clinic,
Box 5605, 114 86 Stockholm, Sweden

Karin Henriksson-Larsén
GIH -The Swedish School of Sport and Health and Sciences
Lidingövägen 1, Box 5626, 114 86 Stockholm, Sweden.

Keywords

Sports injury, athletic injury, accident prevention, recreation, epidemiology

Word count

3 362

Number of references

38

For peer review only

ABSTRACT

Background:

Before preventive actions can be suggested for sports injuries at a national level a solid surveillance system is required in order to study epidemiology, risk factors and mechanisms. There are guidelines for sport injury data collection and classifications in the literature for that purpose. In Sweden, 90% of all athletes, (57/70 sport federations) are insured in the same insurance company and the data from their database could be a foundation for studies on acute sports injuries, at a national level.

Objective:

To evaluate the usefulness of sports injury insurance claims data in sports injury surveillance, at a national level.

Method:

A database with 27947 injuries was exported to an excel file. Access to the corresponding text files was also obtained. Data were reviewed on available information, missing information, and drop-outs. Comparison against ASIDD (Australian Sports Injury Data Dictionary) and with existing Consensus statement in the literature (Football (soccer), Rugby union, Tennis, Cricket and Thoroughbred horse racing) was performed in a structured manner.

Result:

Comparison with ASIDD showed that 93 % of the suggested data items were present in the database, to at least some extent. Compliance with the Consensus statements was generally high (13/18). Almost all claims (83%) contained text information concerning the injury.

Conclusion:

Relatively high quality sports injury data can be obtained from a specific insurance company at national level in Sweden. The database has the potential to be a solid base for research on acute sport injuries in different sports at a national level.

For peer review only

Strengths and limitations of this study:

- Data includes a large material from a database that covers more than 80% of the sports federations in Sweden at a national level.
- The study is a structured examination of the reliability and validity of data, using standardized guidelines from the sport injury literature.
- A limitation is that data is not evaluated for recording rate reliability since there is no national record of sports injuries in the country.

For peer review only

INTRODUCTION

Since approximately 18-30 % of all acute injuries are sports-related[1 2], and almost half these injuries are related to large team sports such as football, handball and basketball [2-4] preventive actions are warranted. Prevention of acute sport injuries requires four stages: (1) identifying the magnitude of the problem (epidemiology), (2) identifying risk factors that may contribute to injuries (etiology), (3) introducing a preventive measure (prevention), and (4) assessing prevention effectiveness by repeating step 1.[5] To obtain comparable data there are guidelines for standardizations of injury definitions and data collection in the literature.[5-10] In football, rugby union, tennis, cricket and thoroughbred horse racing there are Consensus statements on these procedures.[6 10-13]

Most countries do not have a national sports injury surveillance system where injury information from several sports is found in the same database and direct comparisons between sports are difficult to perform (1). Data on sports injury epidemiology may instead be obtained from emergency departments, [2 14] insurance companies, [15-18] other records for specific sports, [19 20] or from a specific competition. [8 21 22] Studies have found that data from insurance companies could be eligible in sport injury research.[16 23 24] Finch (2003) evaluated data from three insurance companies in Australia, compared these data with the desirable and necessary information suggested by ASIDD (The Australian Sports Injury Data Dictionary),[25] and found that relatively high quality sports and recreational injury data can be obtained from insurance claims. [23]

At the time of the present study, 57 out of 70 sport federations under The Swedish Sports Confederation (RF) use the same Swedish insurance company for injury insurance including all organized athletes in each sport federation. This type of insurance covers the athlete for costs associated with their care and treatment after an accidental injury. If the injury results in a medical disability the athlete receives a lump sum in relation to the severity of the disability. In the case of death, a lump sum is paid to the family. All injuries and payments are registered in a database which covers 90% of all athletes in Sweden, at all ages and all levels of sport. Since previous studies have shown that data from insurance companies can be useful in sports injury research, [16 23 26] the aim of this study was to evaluate the use of the database from this particular Swedish insurance company in injury preventive research, and to compare the obtained information with ASIDD and Consensus statements on sport injury surveillance.

For peer review only

Specific aims:

Specific aims of the study were to measure the reliability of the sports injury data by reviewing the injury data collection procedures and a sample of injuries within the database. The validity of the data was examined by comparing the included variables within the database with variables in ASIDD and Consensus statements from the literature on injury definitions and data collection procedures.[6 10-13 23 25] After these evaluations, improvements to the database will be suggested.

METHODS

A sports injury can be registered with the Swedish insurance company within three years from the time the accident occurred. The definition of an injury eligible for an insurance claim is a new accident injury related to sports activities, training or competition organized by the sports federation or sports club and that is reported to the insurance company. The cause of the injury should be a sudden external force and no overuse injuries are registered. Information regarding the injury and claim is routinely kept in a systematic and structured manner within the sports injury database and is updated over time as new information is available. The majority of the diagnoses are made by doctors. From the database, details can be transformed into an Excel file for statistical analysis. The insurance company performs their own audit of the administrators' claim processing. Randomly selected injury claims, a percentage of the total number of claims, are reviewed by senior administrators every month. Once a year the company auditors also undertake an extensive review of randomly selected injury claims. The latest audit on sports injury claims was performed in the autumn of 2011, and concluded that claim settlements were generally performed with high quality and accuracy.

Randomly selected settled injury claims between 2006 and 2010 including 27 947 unique injuries was exported to an Excel file, and access to a local database for text information surrounding the injury, was received from the insurance company. Data included information on sports activity, age, gender, residence at time of injury, date of injury, diagnosis code (type of injury, injured body region and body part, left or right side of body), and type of financial compensation (medical treatment, dental treatment, permanent disability or death). The data also contained information on disability assessment, the administrator, and costs. The local database contained text files with descriptions of injury mechanisms, training or competition, surface and environment, and protective equipment. The local database also included information on the administrator´s procedure.

For peer review only

To ensure data reliability, the database was reviewed for available and missing information based on desirable and necessary information previously defined in sports injury research. [6 10-13 25] From the 27 947 unique injuries, 310 random samples were chosen to evaluate data consistency and lack of information within the Excel file compared to text files in the local database. The insurance company's claim and documentation procedures were studied. All administrators, all sports, and all ages of athletes were represented in the randomly selected samples. Data validity was examined both by comparing the injury data with ASIDD guidelines, in order to make comparisons between different sports [25], and it was also examined relative to Consensus statements in specific sports.[6 10-13]

The Australian Sports Injury Data Dictionary, ASIDD (1997) provide standardized guidelines for injury data collection .[25] According to ASIDD "*Core"* items (category 1) should be presented in all sports injury data collections, "*Strongly recommended"* items (category 2) should be included, where possible, to give detailed additional injury information and "*Recommended"* items (category 3) can provide further data pertaining to injury circumstances (Table 1). [23] Comparison of the Swedish insurance company data with ASIDD was performed, in the same way as earlier performed by Finch in Australia.[23] The presentation of items was registered as "yes" (present in the database), "partially" (partially present in the database) and "no" (not present in the database). A scoring system was used to assess the amount of information in the insurance claims according to ASIDD guidelines:

1 Data item fully present (should be fully coded according to ASIDD guidelines)

0 Data item totally absent (in ASIDD but not in the insurance database)

0.5 Data item partially present (some, but not all, of the details specified in ASIDD were in the database)

In order to ensure the validity of the data for specific sports, a comparison with five existing Consensus statements on injury definitions and data collection procedures in the literature [6 10-13] was performed.

Statistics

The computer programs Microsoft office Excel 2007 and SPSS (SPSS for Windows, version 21.0) were used. Reliability of the claims and documentation procedures are expressed as a percentage by dividing the number of missing values with the number of reviewed posts. When validating the insurance data with ASIDD, a data category agreement score was calculated as the sum of the individual scored items. An overall agreement score calculated as the sum of the three data category agreements to determine the general quality of information.

For peer review only

[23] Validation of the data using Consensus statements was based on the established information criteria published in the scientific literature for football, rugby union, tennis, cricket and thoroughbred horse racing. [6 10-13]

Ethical approval

The project is a part of a more extensive investigation which has been approved by the Regional Ethical Committee in Stockholm (Dnr 2012/1436-31/1). Data was decoded from identification numbers, and only the research leaders have access to the data. No individual consent of the athletes has been requested as results will be presented on a group level and the insurance policy also informs the policyholders that data can be used in research.

RESULTS

Loss of data

Excel file

Lack of a valid social security number occurred in 103 of 27 947 posts (0,4%) in the Excel file. This was due to foreign athletes practicing their sport in Sweden without a Swedish social security number. The gender and age of these athletes can be determined from the text files in the local database. Fifty-five of 27 947 injuries (0.2%) lacked a diagnosis code but all codes could be identified when reading the texts in the database. Residence at the time of injury (postal code) was missing in approximately 23 % of the posts.

Local database (text files)

Text files in the local database were available for more than 80 % of the 310 random samples taken from the total number of claims. From 2011 onwards, text files were presented in almost all the claims. In some claims from 2007 and earlier, the texts were not always in the local database but could be ordered in hard copy format, which are not included in this study. The diagnosis codes were consistent in more than 88 % of the samples. Incorrect diagnoses were found in three samples (1%), unknown diagnoses in six samples (2%), and correct location but incorrect injury type in 13 samples (4%).

An evaluation of different disciplines within the sport federation was also performed. For example, the Swedish Ski Association has different disciplines within the sport, and in the database there are different category numbers for alpine skiing, cross country skiing, snowboarding etc. The Swedish Mixed Martial Arts Federation and the Swedish Motorsport Federation also have different disciplines within the sport, and thus different category numbers in the data base. All samples tested were found to be in the correct sport, however

For peer review only

consistency between the category number in the Excel file and the discipline described in the text file in the local database was approximately 83%.

Compliance to ASIDD

Comparison with ASIDD showed that 93 % (i.e. 28 of 30) of the suggested data items were present, to at least some extent. The overall agreement score was 24 out of a highest possible score of 30 (Table 1). Within "Core" ASIDD items, no missing data were found. All "strongly recommended" items were present in some form but full information was missing in four of them (Table 1). In "recommended" items, five out of eight were present but three of them did not include full information (Table 1). Part of the information regarding ASIDD items was found in the text files within the local database and not in the Excel file.

Table 1. Comparison of the insurance database information with the ASIDD recommendations.

**ASIDD "Core" data item**	**In the insurance data files**
Date of injury	yes
Age	yes
Gender	yes
Activity when injured - broad areas	yes
Mechanism of injury	yes
Body region and body chart	yes
Nature of injury	yes
**Category agreement score** (maximum score = 7)	7 (100%)
**ASIDD "Strongly Recommended" data item**	**In the insurance data files**
Person recording case information	yes
Immediate source of injury record	yes
Area of usual residence	yes
Name of injury place - text	yes
Place of injury - type	yes
Sport and recreation places - specific	yes
Activity when injured -name of sport or activity	yes
Injury factors	yes
Equipment used with intent to protect against injury	partially
Narrative of mechanism of injury	yes
Date of presentation	yes
Treatment	partially
Advice given to injured person	partially
Referral	partially
Treating person	yes
**Category agreement score** (maximum score = 15)	13 (87%)
**ASIDD "Recommended" data item**	**In the insurance data files**
Time of injury	no
Date of injury record	yes
Part of specific injury place	partially
Phase or aspect of involvement in activity	partially
Grade/level of play	partially
Specific structure injured	partially
Time of presentation	no
Reason for presentation	yes
**Category agreement score** (maximum score = 8)	4 (50%)

For peer review only

Compliance with Consensus statements

Compliance with required information in the Consensus statements, published in sports-related scientific literature, were high (13 of 18 items) except for body mass, height, dominant leg/arm, position of play, and date of returning to sport after injury (Table 2). Some differences in desirable information were found among the different sport federations. Information from seven items existed in the insurance database in some, but not all, claims (Table 2).

For peer review only

Table 2. Comparison between the information from the insurance database with required information in the Consensus statements.

Possible items	Football	Rugby	Tennis	Cricket	Thoroughbred horse racing	Insurance data
Age	x	x	x	x	x	x
Gender	x	x	x	x	x	x
Body mass	x	x	x		x	
Height	x	x	x		x	
Dominant leg/arm	x		x			
Standard/Level of play		x	x		x	x -
Position in play	x	x		x		
Date of onset	x	x	x	x	x	x
Injury diagnosis/Nature of injury	x	x	x	x	x	x
Injured side	x	x	x	x	x	x
Match /training	x	x	x	x	x	x-
Circumstances surrounding the injury	x	x	x	x	x	x -
Date of return to sport	x	x	x		x	
Mechanisms	x	x	x	x	x	x -
Free text (around the injury)	x		x			x -
Protective equipment					x	x -
Surface (playing ground)			x		x	x -
Treatment (medical)				x	x	x -

x - data exist in some but not in all claims

DISCUSSION

Most countries do not have a national sports injury surveillance system where sport injury information from several sports is found in the same database, but in the US there are national sports injury surveillance systems for adolescent high school and collegiate athletes. [27 28]Thus, insurance data is a possible way of obtaining sports injury information.[23]This study confirms that relatively high quality sports injury data can be obtained from a sports injury database within a specific insurance company in Sweden. The database included all athletes, in all ages and at different training and competing levels, in 57 sports federations.

In sports injury research, epidemiology and etiology are essential as a foundation for sport injury prevention.[5] It is well known that variations in definitions and methodologies create differences in the results and conclusions from studies of sports injuries.[5 26 29] It could therefore be difficult to compare results from different studies. The database in this study includes all licensed and unlicensed athletes in 57 sports federations and the definitions and data collection procedures are the same regardless of sport, thus comparisons between sports, gender and ages are easily made. Since the database only includes accidental injuries and no data of overuse injuries is available, it will not cover the whole injury panorama in each sport.

Earlier studies have shown that insurance claims have the potential to provide detailed information about sports injuries, the records are routinely kept in a systematic manner, and comparisons between data can easily be made.[16 23 26] Different studies on sports injuries have used insurance data for different sports, such as netball, rugby and football.[16-18 24] One smaller study in Finland compared injury profiles in football, ice hockey, volleyball, basketball, judo and karate using national insurance register data.[30] Insurance databases have historically been invaluable for guiding road and workplace safety.[31-33] A recent published study on administrative databases, including insurance databases, concluded that they could be a robust research tool. They allow for longitudinal designs, incidence calculation, and large sample size and power, even for rare events.[26]

The insurance data in the present study included many of the ASIDD items; all “core” and “strongly recommended” items and more than 60% of the “recommended” items were present. [25] Data items that, according to ASIDD, could be improved, related to treatment, referral and advice given to the athlete when injured.[25] Precise and specific information about injury location, phase or aspect of activity, equipment used and specific structure injured was also missing.[25] One detail not registered was the grade or level of play, although it could occasionally be read in the text file that the athlete competed in a national championship.

The results from comparing the injury claim data with desirable information in the Consensus statements [6 10-13] show that the insurance database could be useful in research on sport injuries. However, missing data on personal information such as height, body mass, dominant leg/arm, position of play, and return to play are important in some research. Information about return to play is often used to calculate the absence of sporting activity and hence the severity

For peer review only

of the injury.[5] In the insurance data, injury type and level of permanent medical disability are two methods of grading injury severity. The definition of medical disability is set by "Insurance Sweden" (the industry organization for insurance companies in Sweden). http://www.svenskforsakring.se/Global/Invaliditetsintyg/Medicinsk_invaliditet_skador120911.pdf

A recent published consensus statement for injury surveillance in Athletics is based on previous consensus statements [6 10-13] on details of athlete baseline and recordable incident/injury information. It also reflects more profound information on the specific issues facing Athletics at international and domestic levels.[34]

Prospective data collection procedure is considered to be the most appropriate method for sport injury surveillance.[6 10-13] When using insurance claims data, both retrospectively and prospectively study design can be used, depending on the starting point of the study. Though, some of the claims may be reported to the insurance company retrospectively since the athlete can report an injury up to three years after the accident or in rare cases at the time when the athlete gained knowledge that injury may be reported to the insurance company (up to ten year after injury). In the present study where the data was retrospectively analyzed, injuries in the database were reported, in median 16 days from the day they occurred.

Van Mechelen suggests that an adequate sports injury surveillance system should be sensitive enough to answer "how many, how often, how long, and serious?".[35] If these questions are to be answered on a national level, where the magnitude of sports injury problems are investigated by comparing incidence rates between various sports, or detecting trends and changes over time, a system that can detect large numbers of cases is needed.[35] The system should be as simple and unambiguous as possible and should at least contain a universally applicable definition of sports injury, of sports participation and consensus on population at risk and time at risk.[35] The insurance data in this study are contained in a national register which covers all athletes in more than 80% of all sport federations, and includes, for example, sports with many athletes such as football, ice hockey, handball, floorball, golf, material arts and motor sports.

Lack of full information within some items both compared with ASIDD and the Consensus statements suggests that the insurance data could be improved upon in the future with the collection of additional details and standardizations of data variables. Some of these changes are implemented in the present insurance database as the result of this study. For example, by using headlines in the database, the terminology regarding injury mechanisms and injury

For peer review only

circumstances, match/training, surface and free text around the injury have been standardized based on desirable sport injury information in the literature. [6 10-13 25] This, enhance the possibility for future prospective studies of purpose-design surveillance at national level, with a large number of athletes and long follow up time.

Missing desirable information in the insurance data, including total number of athletes in each sport and exposure to sport, could be a weakness when performing epidemiological studies on sport injuries.[5 36] In further studies using data from the insurance company, assessment of sports injury incidence rate (the number of new sports injuries / population at risk over time) or sports injury prevalence (the proportion of injured athlete in the population at risk at any one time) may be calculated by using information from the specific sports federation regarding the number of participants and licensed athletes in each sport.[35 37] When conducting studies on exposure related incidence of injury an estimation of exposure to sport must be performed, for example by using estimated information from the specific sports federations regarding time spent on training and competition for each age group and standard/level of sport.

Despite the quality of the sports injury surveillance system, one will always be confronted with some sort of bias, recall bias, over- or underestimation of sports participation, incomplete responses, non-response, invalid injury description etc.[35] The insurance data collection is structured and routinely kept in a systematic manner, and the insurance company works to keep the data accurate and truthful. The database population consists of athletes who *report* an injury to the insurance company and therefore the data it contains likely underestimates the number of injuries. A study from 2005 on anterior cruciate ligament injuries in football, floorball, handball and ice hockey used the same insurance data as the present study and compared it to two medical databases to determine recording rate reliability.[18] The result showed that 74% of all injuries sustained were recorded in the insurance database.[18] Roos et al (1995) also studying the same database estimated that 98% of the football injuries were registered within seven years.[38] The amount of financial compensation for each claim from the insurance company may influence the rate of injury registration. More severe injuries that require medical treatment or may result in a permanent medical disability are more likely to be reported, as well as dental injuries where treatment is expensive. The athlete can report the injury within three years after the accident, and medical disabilities are assessed in one to two

For peer review only

years after the accident occurred. This may result in a delay of recording in the database which must be considered when analyzing the data.[38]

Despite these weaknesses, the data are important for the detection of serious injuries and injury mechanisms in different sports at national level. Injury data, such as incidence and severity of injury can be compared between sports, gender and ages and be followed over time, for example in longitudinal studies of injury prevention measures at a national level.

CONCLUSION

Before preventative actions can be suggested, sports injury epidemiology, risk factors and injury mechanisms need to be compiled. Since a comprehensive national database from a Swedish insurance company contains most of the desirable information in sport injury research, and further improvements to the database information have been implemented, this database now has the potential to be a base for research on acute (accidental) injuries in organized sports at a national level. It could also constitute a foundation for assessment of sport injury prevention at a national level.

Funding

This work was supported by Folksam, a Swedish insurance company

Contributorship statement

The corresponding author and co-authors have all substantial contributed to the research and to the final document by intellectual content, conception and design, analysis and interpretation of data as well as drafting and critical revision of the manuscript.

Data sharing statement

No additional data available.

Competing Interests

None declared

For peer review only

REFERENCES

1. Caine D, Caine C, Maffulli N. Incidence and distribution of pediatric sport-related injuries. Clin J Sport Med 2006;**16**(6):500-13 doi: 10.1097/01.jsm.0000251181.36582.a0[published Online First: Epub Date]|.
2. Conn JM, Annest JL, Gilchrist J. Sports and recreation related injury episodes in the US population, 1997-99. Inj Prev 2003;**9**(2):117-23
3. (EuroSafe) EAfIPaSP. Injuries in the European Union. Summary of injury statisticsfor the years 2008-2010. 2013(4)
4. Knowles SB, Marshall SW, Bowling JM, et al. A prospective study of injury incidence among North Carolina high school athletes. Am J Epidemiol 2006;**164**(12):1209-21 doi: 10.1093/aje/kwj337[published Online First: Epub Date]|.
5. van Mechelen W, Hlobil H, Kemper HC. Incidence, severity, aetiology and prevention of sports injuries. A review of concepts. Sports Med 1992;**14**(2):82-99
6. Fuller CW, Ekstrand J, Junge A, et al. Consensus statement on injury definitions and data collection procedures in studies of football (soccer) injuries. Clin J Sport Med 2006;**16**(2):97-106
7. Finch C. A new framework for research leading to sports injury prevention. J Sci Med Sport 2006;**9**(1-2):3-9; discussion 10 doi: 10.1016/j.jsams.2006.02.009[published Online First: Epub Date]|.
8. Junge A, Engebretsen L, Alonso JM, et al. Injury surveillance in multi-sport events: the International Olympic Committee approach. Br J Sports Med 2008;**42**(6):413-21 doi: 10.1136/bjsm.2008.046631[published Online First: Epub Date]|.
9. Hammond LE, Lilley J, Ribbans WJ. Coding sports injury surveillance data: has version 10 of the Orchard Sports Injury Classification System improved the classification of sports medicine diagnoses? Br J Sports Med 2009;**43**(7):498-502 doi: 10.1136/bjsm.2008.051979[published Online First: Epub Date]|.
10. Turner M, Fuller CW, Egan D, et al. European consensus on epidemiological studies of injuries in the thoroughbred horse racing industry. Br J Sports Med 2012;**46**(10):704-8 doi: 10.1136/bjsports-2011-090312[published Online First: Epub Date]|.
11. Fuller CW, Molloy MG, Bagate C, et al. Consensus statement on injury definitions and data collection procedures for studies of injuries in rugby union. Clin J Sport Med 2007;**17**(3):177-81 doi: 10.1097/JSM.0b013e31803220b3[published Online First: Epub Date]|.
12. Pluim BM, Fuller CW, Batt ME, et al. Consensus statement on epidemiological studies of medical conditions in tennis, April 2009. Br J Sports Med 2009;**43**(12):893-7 doi: 10.1136/bjsm.2009.064915[published Online First: Epub Date]|.
13. Orchard J, Newman D, Stretch R, et al. Methods for injury surveillance in international cricket. J Sci Med Sport 2005;**8**(1):1-14
14. Nelson NG, McKenzie LB. Mountain biking-related injuries treated in emergency departments in the United States, 1994-2007. Am J Sports Med 2011;**39**(2):404-9 doi: 10.1177/0363546510383478[published Online First: Epub Date]|.
15. de Loes M, Dahlstedt LJ, Thomee R. A 7-year study on risks and costs of knee injuries in male and female youth participants in 12 sports. Scand J Med Sci Sports 2000;**10**(2):90-7
16. Otago L, Peake J. The role of insurance data in setting priorities for netball injury prevention strategies. J Sci Med Sport 2007;**10**(2):105-9 doi: 10.1016/j.jsams.2006.05.016[published Online First: Epub Date]|.
17. Soderman K, Pietila T, Alfredson H, et al. Anterior cruciate ligament injuries in young females playing soccer at senior levels. Scand J Med Sci Sports 2002;**12**(2):65-8

For peer review only

18. Forssblad M, Weidenhielm L, Werner S. Knee surgery costs in football, floor ball, European team handball and ice hockey. Scand J Med Sci Sports 2005;**15**(1):43-7 doi: 10.1111/j.1600-0838.2004.00392.x[published Online First: Epub Date]|.
19. Westin M, Alricsson M, Werner S. Injury profile of competitive alpine skiers: a five-year cohort study. Knee Surg Sports Traumatol Arthrosc 2012;**20**(6):1175-81 doi: 10.1007/s00167-012-1921-x[published Online First: Epub Date]|.
20. Junge A, Dvorak J. Injury surveillance in the World Football Tournaments 1998-2012. Br J Sports Med 2013;**47**(12):782-8 doi: 10.1136/bjsports-2013-092205[published Online First: Epub Date]|.
21. Engebretsen L, Soligard T, Steffen K, et al. Sports injuries and illnesses during the London Summer Olympic Games 2012. Br J Sports Med 2013;**47**(7):407-14 doi: 10.1136/bjsports-2013-092380[published Online First: Epub Date]|.
22. Engebretsen L, Steffen K, Alonso JM, et al. Sports injuries and illnesses during the Winter Olympic Games 2010. Br J Sports Med 2010;**44**(11):772-80 doi: 10.1136/bjsm.2010.076992[published Online First: Epub Date]|.
23. Finch C. How useful are insurance claim data for sports injury prevention purposes? Inj Control Saf Promot. 2003/07/17 ed, 2003:181-3.
24. King DA, Hume PA, Milburn P, et al. Rugby league injuries in New Zealand: a review of 8 years of Accident Compensation Corporation injury entitlement claims and costs. Br J Sports Med 2009;**43**(8):595-602 doi: 10.1136/bjsm.2009.061481[published Online First: Epub Date]|.
25. Party TASIDW. Australian Sports Injury Data Dictionary. Guidelines for injury data collection and classification for the prevention and control of injury in sport and recreation., 1997.
26. Gavrielov-Yusim N, Friger M. Use of administrative medical databases in population-based research. J Epidemiol Community Health 2013 doi: 10.1136/jech-2013-202744[published Online First: Epub Date]|.
27. Kucera KL, Marshall SW, Bell DR, et al. Validity of soccer injury data from the National Collegiate Athletic Association's Injury Surveillance System. J Athl Train 2011;**46**(5):489-99
28. Robinson TW, Corlette J, Collins CL, et al. Shoulder injuries among US high school athletes, 2005/2006-2011/2012. Pediatrics 2014;**133**(2):272-9 doi: 10.1542/peds.2013-2279[published Online First: Epub Date]|.
29. Finch CF. An overview of some definitional issues for sports injury surveillance. Sports Med 1997;**24**(3):157-63
30. Kujala UM, Taimela S, Antti-Poika I, et al. Acute injuries in soccer, ice hockey, volleyball, basketball, judo, and karate: analysis of national registry data. BMJ 1995;**311**(7018):1465-8
31. Badii M, Keen D, Yu S, et al. Evaluation of a comprehensive integrated workplace-based program to reduce occupational musculoskeletal injury and its associated morbidity in a large hospital. Journal of occupational and environmental medicine / American College of Occupational and Environmental Medicine 2006;**48**(11):1159-65 doi: 10.1097/01.jom.0000243416.18334.c8[published Online First: Epub Date]|.
32. McCall BP, Horwitz IB, Taylor OA. Occupational eye injury and risk reduction: Kentucky workers' compensation claim analysis 1994-2003. Inj Prev 2009;**15**(3):176-82 doi: 10.1136/ip.2008.020024[published Online First: Epub Date]|.
33. Krafft M, Kullgren A, Lie A, et al. The use of seat belts in cars with smart seat belt reminders--results of an observational study. Traffic Inj Prev 2006;**7**(2):125-9 doi: 10.1080/15389580500509278[published Online First: Epub Date]|.

For peer review only

34. Timpka T, Alonso JM, Jacobsson J, et al. Injury and illness definitions and data collection procedures for use in epidemiological studies in Athletics (track and field): consensus statement. Br J Sports Med 2014;**48**(7):483-90 doi: 10.1136/bjsports-2013-093241[published Online First: Epub Date]|.
35. van Mechelen W. Sports injury surveillance systems. 'One size fits all'? Sports Med 1997;**24**(3):164-8
36. de Loes M. Exposure data. Why are they needed? Sports Med 1997;**24**(3):172-5
37. Brooks JH, Fuller CW. The influence of methodological issues on the results and conclusions from epidemiological studies of sports injuries: illustrative examples. Sports Med 2006;**36**(6):459-72
38. Roos H, Ornell M, Gardsell P, et al. Soccer after anterior cruciate ligament injury--an incompatible combination? A national survey of incidence and risk factors and a 7-year follow-up of 310 players. Acta orthopaedica Scandinavica 1995;**66**(2):107-12

For peer review only

Title

Insurance claims data - a possible solution for a national sports injury surveillance system? – An evaluation of data information against ASIDD and Consensus statements on sports injury surveillance.

Corresponding author

Malin Åman

GIH -The Swedish School of Sport and Health and Sciences

Lidingövägen 1, Box 5626, 114 86 Stockholm, Sweden.

malin.aman@gih.se

telephone +46739947349, fax +46812053880

Co-authors

Magnus Forssblad

Department of Molecular Medicine and Surgery, Karolinska Institut,

Stockholm Sports Trauma Research Center, Capio Artro Clinic,

Box 5605, 114 86 Stockholm, Sweden

Karin Henriksson-Larsén

GIH -The Swedish School of Sport and Health and Sciences

Lidingövägen 1, Box 5626, 114 86 Stockholm, Sweden.

Keywords

Sports injury, athletic injury, accident prevention, recreation, epidemiology

Word count

3 362

Number of references

38

For peer review only

ABSTRACT

Background:

Before preventive actions can be suggested for sports injuries at a national level a solid surveillance system is required in order to study epidemiology, risk factors and mechanisms. There are guidelines for sport injury data collection and classifications in the literature for that purpose. In Sweden, 90% of all athletes, (57/70 sport federations) are insured in the same insurance company and the data from their database could be a foundation for studies on acute sports injuries, at a national level.

Objective:

To evaluate the usefulness of sports injury insurance claims data in sports injury surveillance, at a national level.

Method:

A database with 27947 injuries was exported to an excel file. Access to the corresponding text files was also obtained. Data were reviewed on available information, missing information, and drop-outs. Comparison against ASIDD (Australian Sports Injury Data Dictionary) and with existing Consensus statement in the literature (Football (soccer), Rugby union, Tennis, Cricket and Thoroughbred horse racing) was performed in a structured manner.

Result:

Comparison with ASIDD showed that 93 % of the suggested data items were present in the database, to at least some extent. Compliance with the Consensus statements was generally high (13/18). Almost all claims (83%) contained text information concerning the injury.

Conclusion:

Relatively high quality sports injury data can be obtained from a specific insurance company at national level in Sweden. The database has the potential to be a solid base for research on acute sport injuries in different sports at a national level.

For peer review only

Strengths and limitations of this study:

- Data includes a large material from a database that covers more than 80% of the sports federations in Sweden at a national level.
- The study is a structured examination of the reliability and validity of data, using standardized guidelines from the sport injury literature.
- A limitation is that data is not evaluated for recording rate reliability since there is no national record of sports injuries in the country.

For peer review only

INTRODUCTION

Since approximately 18-30 % of all acute injuries are sports-related[1 2], and almost half these injuries are related to large team sports such as football, handball and basketball [2-4] preventive actions are warranted. Prevention of acute sport injuries requires four stages: (1) identifying the magnitude of the problem (epidemiology), (2) identifying risk factors that may contribute to injuries (etiology), (3) introducing a preventive measure (prevention), and (4) assessing prevention effectiveness by repeating step 1.[5] To obtain comparable data there are guidelines for standardizations of injury definitions and data collection in the literature.[5-10] In football, rugby union, tennis, cricket and thoroughbred horse racing there are Consensus statements on these procedures.[6 10-13]

Most countries do not have a national sports injury surveillance system where injury information from several sports is found in the same database and direct comparisons between sports are difficult to perform (1). Data on sports injury epidemiology may instead be obtained from emergency departments, [2 14] insurance companies, [15-18] other records for specific sports, [19 20] or from a specific competition. [8 21 22] Studies have found that data from insurance companies could be eligible in sport injury research.[16 23 24] Finch (2003) evaluated data from three insurance companies in Australia, compared these data with the desirable and necessary information suggested by ASIDD (The Australian Sports Injury Data Dictionary),[25] and found that relatively high quality sports and recreational injury data can be obtained from insurance claims. [23]

At the time of the present study, 57 out of 70 sport federations under The Swedish Sports Confederation (RF) use the same Swedish insurance company for injury insurance including all organized athletes in each sport federation. This type of insurance covers the athlete for costs associated with their care and treatment after an accidental injury. If the injury results in a medical disability the athlete receives a lump sum in relation to the severity of the disability. In the case of death, a lump sum is paid to the family. All injuries and payments are registered in a database which covers 90% of all athletes in Sweden, at all ages and all levels of sport. Since previous studies have shown that data from insurance companies can be useful in sports injury research, [16 23 26] the aim of this study was to evaluate the use of the database from this particular Swedish insurance company in injury preventive research, and to compare the obtained information with ASIDD and Consensus statements on sport injury surveillance.

For peer review only

Specific aims:

Specific aims of the study were to measure the reliability of the sports injury data by reviewing the injury data collection procedures and a sample of injuries within the database. The validity of the data was examined by comparing the included variables within the database with variables in ASIDD and Consensus statements from the literature on injury definitions and data collection procedures.[6 10-13 23 25] After these evaluations, improvements to the database will be suggested.

METHODS

A sports injury can be registered with the Swedish insurance company within three years from the time the accident occurred. The definition of an injury eligible for an insurance claim is a new accident injury related to sports activities, training or competition organized by the sports federation or sports club and that is reported to the insurance company. The cause of the injury should be a sudden external force and no overuse injuries are registered. Information regarding the injury and claim is routinely kept in a systematic and structured manner within the sports injury database and is updated over time as new information is available. The majority of the diagnoses are made by doctors. From the database, details can be transformed into an Excel file for statistical analysis. The insurance company performs their own audit of the administrators' claim processing. Randomly selected injury claims, a percentage of the total number of claims, are reviewed by senior administrators every month. Once a year the company auditors also undertake an extensive review of randomly selected injury claims. The latest audit on sports injury claims was performed in the autumn of 2011, and concluded that claim settlements were generally performed with high quality and accuracy.

Randomly selected settled injury claims between 2006 and 2010 including 27 947 unique injuries was exported to an Excel file, and access to a local database for text information surrounding the injury, was received from the insurance company. Data included information on sports activity, age, gender, residence at time of injury, date of injury, diagnosis code (type of injury, injured body region and body part, left or right side of body), and type of financial compensation (medical treatment, dental treatment, permanent disability or death). The data also contained information on disability assessment, the administrator, and costs. The local database contained text files with descriptions of injury mechanisms, training or competition, surface and environment, and protective equipment. The local database also included information on the administrator´s procedure.

For peer review only

To ensure data reliability, the database was reviewed for available and missing information based on desirable and necessary information previously defined in sports injury research. [6 10-13 25] From the 27 947 unique injuries, 310 random samples were chosen to evaluate data consistency and lack of information within the Excel file compared to text files in the local database. The insurance company's claim and documentation procedures were studied. All administrators, all sports, and all ages of athletes were represented in the randomly selected samples. Data validity was examined both by comparing the injury data with ASIDD guidelines, in order to make comparisons between different sports [25], and it was also examined relative to Consensus statements in specific sports.[6 10-13]

The Australian Sports Injury Data Dictionary, ASIDD (1997) provide standardized guidelines for injury data collection .[25] According to ASIDD "*Core"* items (category 1) should be presented in all sports injury data collections, "*Strongly recommended"* items (category 2) should be included, where possible, to give detailed additional injury information and "*Recommended"* items (category 3) can provide further data pertaining to injury circumstances (Table 1). [23] Comparison of the Swedish insurance company data with ASIDD was performed, in the same way as earlier performed by Finch in Australia.[23] The presentation of items was registered as "yes" (present in the database), "partially" (partially present in the database) and "no" (not present in the database). A scoring system was used to assess the amount of information in the insurance claims according to ASIDD guidelines:

1 Data item fully present (should be fully coded according to ASIDD guidelines)

0 Data item totally absent (in ASIDD but not in the insurance database)

0.5 Data item partially present (some, but not all, of the details specified in ASIDD were in the database)

In order to ensure the validity of the data for specific sports, a comparison with five existing Consensus statements on injury definitions and data collection procedures in the literature [6 10-13] was performed.

Statistics

The computer programs Microsoft office Excel 2007 and SPSS (SPSS for Windows, version 21.0) were used. Reliability of the claims and documentation procedures are expressed as a percentage by dividing the number of missing values with the number of reviewed posts. When validating the insurance data with ASIDD, a data category agreement score was calculated as the sum of the individual scored items. An overall agreement score calculated as the sum of the three data category agreements to determine the general quality of information.

For peer review only

[23] Validation of the data using Consensus statements was based on the established information criteria published in the scientific literature for football, rugby union, tennis, cricket and thoroughbred horse racing. [6 10-13]

Ethical approval

The project is a part of a more extensive investigation which has been approved by the Regional Ethical Committee in Stockholm (Dnr 2012/1436-31/1). Data was decoded from identification numbers, and only the research leaders have access to the data. No individual consent of the athletes has been requested as results will be presented on a group level and the insurance policy also informs the policyholders that data can be used in research.

RESULTS

Loss of data

Excel file

Lack of a valid social security number occurred in 103 of 27 947 posts (0,4%) in the Excel file. This was due to foreign athletes practicing their sport in Sweden without a Swedish social security number. The gender and age of these athletes can be determined from the text files in the local database. Fifty-five of 27 947 injuries (0.2%) lacked a diagnosis code but all codes could be identified when reading the texts in the database. Residence at the time of injury (postal code) was missing in approximately 23 % of the posts.

Local database (text files)

Text files in the local database were available for more than 80 % of the 310 random samples taken from the total number of claims. From 2011 onwards, text files were presented in almost all the claims. In some claims from 2007 and earlier, the texts were not always in the local database but could be ordered in hard copy format, which are not included in this study. The diagnosis codes were consistent in more than 88 % of the samples. Incorrect diagnoses were found in three samples (1%), unknown diagnoses in six samples (2%), and correct location but incorrect injury type in 13 samples (4%).

An evaluation of different disciplines within the sport federation was also performed. For example, the Swedish Ski Association has different disciplines within the sport, and in the database there are different category numbers for alpine skiing, cross country skiing, snowboarding etc. The Swedish Mixed Martial Arts Federation and the Swedish Motorsport Federation also have different disciplines within the sport, and thus different category numbers in the data base. All samples tested were found to be in the correct sport, however

For peer review only

consistency between the category number in the Excel file and the discipline described in the text file in the local database was approximately 83%.

Compliance to ASIDD

Comparison with ASIDD showed that 93 % (i.e. 28 of 30) of the suggested data items were present, to at least some extent. The overall agreement score was 24 out of a highest possible score of 30 (Table 1). Within "Core" ASIDD items, no missing data were found. All "strongly recommended" items were present in some form but full information was missing in four of them (Table 1). In "recommended" items, five out of eight were present but three of them did not include full information (Table 1). Part of the information regarding ASIDD items was found in the text files within the local database and not in the Excel file.

Table 1. Comparison of the insurance database information with the ASIDD recommendations.

**ASIDD "Core" data item**	**In the insurance data files**
Date of injury	yes
Age	yes
Gender	yes
Activity when injured - broad areas	yes
Mechanism of injury	yes
Body region and body chart	yes
Nature of injury	yes
**Category agreement score** (maximum score = 7)	7 (100%)
**ASIDD "Strongly Recommended" data item**	**In the insurance data files**
Person recording case information	yes
Immediate source of injury record	yes
Area of usual residence	yes
Name of injury place - text	yes
Place of injury - type	yes
Sport and recreation places - specific	yes
Activity when injured -name of sport or activity	yes
Injury factors	yes
Equipment used with intent to protect against injury	partially
Narrative of mechanism of injury	yes
Date of presentation	yes
Treatment	partially
Advice given to injured person	partially
Referral	partially
Treating person	yes
**Category agreement score** (maximum score = 15)	13 (87%)
**ASIDD "Recommended" data item**	**In the insurance data files**
Time of injury	no
Date of injury record	yes
Part of specific injury place	partially
Phase or aspect of involvement in activity	partially
Grade/level of play	partially
Specific structure injured	partially
Time of presentation	no
Reason for presentation	yes
**Category agreement score** (maximum score = 8)	4 (50%)

For peer review only

Compliance with Consensus statements

Compliance with required information in the Consensus statements, published in sports-related scientific literature, were high (13 of 18 items) except for body mass, height, dominant leg/arm, position of play, and date of returning to sport after injury (Table 2). Some differences in desirable information were found among the different sport federations. Information from seven items existed in the insurance database in some, but not all, claims (Table 2).

Table 2. Comparison between the information from the insurance database with required information in the Consensus statements.

Possible items	Football	Rugby	Tennis	Cricket	Thoroughbred horse racing	Insurance data
Age	x	x	x	x	x	x
Gender	x	x	x	x	x	x
Body mass	x	x	x		x	
Height	x	x	x		x	
Dominant leg/arm	x		x			
Standard/Level of play		x	x		x	x -
Position in play	x	x		x		
Date of onset	x	x	x	x	x	x
Injury diagnosis/Nature of injury	x	x	x	x	x	x
Injured side	x	x	x	x	x	x
Match /training	x	x	x	x	x	x-
Circumstances surrounding the injury	x	x	x	x	x	x -
Date of return to sport	x	x	x		x	
Mechanisms	x	x	x	x	x	x -
Free text (around the injury)	x		x			x -
Protective equipment					x	x -
Surface (playing ground)			x		x	x -
Treatment (medical)				x	x	x -

x - data exist in some but not in all claims

DISCUSSION

Most countries do not have a national sports injury surveillance system where sport injury information from several sports is found in the same database, but in the US there are national sports injury surveillance systems for adolescent high school and collegiate athletes. [27 28]Thus, insurance data is a possible way of obtaining sports injury information.[23]This study confirms that relatively high quality sports injury data can be obtained from a sports injury database within a specific insurance company in Sweden. The database included all athletes, in all ages and at different training and competing levels, in 57 sports federations.

For peer review only

In sports injury research, epidemiology and etiology are essential as a foundation for sport injury prevention.[5] It is well known that variations in definitions and methodologies create differences in the results and conclusions from studies of sports injuries.[5 26 29] It could therefore be difficult to compare results from different studies. The database in this study includes all licensed and unlicensed athletes in 57 sports federations and the definitions and data collection procedures are the same regardless of sport, thus comparisons between sports, gender and ages are easily made. Since the database only includes accidental injuries and no data of overuse injuries is available, it will not cover the whole injury panorama in each sport.

Earlier studies have shown that insurance claims have the potential to provide detailed information about sports injuries, the records are routinely kept in a systematic manner, and comparisons between data can easily be made.[16 23 26] Different studies on sports injuries have used insurance data for different sports, such as netball, rugby and football.[16-18 24] One smaller study in Finland compared injury profiles in football, ice hockey, volleyball, basketball, judo and karate using national insurance register data.[30] Insurance databases have historically been invaluable for guiding road and workplace safety.[31-33] A recent published study on administrative databases, including insurance databases, concluded that they could be a robust research tool. They allow for longitudinal designs, incidence calculation, and large sample size and power, even for rare events.[26]

The insurance data in the present study included many of the ASIDD items; all “core” and “strongly recommended” items and more than 60% of the “recommended” items were present. [25] Data items that, according to ASIDD, could be improved, related to treatment, referral and advice given to the athlete when injured.[25] Precise and specific information about injury location, phase or aspect of activity, equipment used and specific structure injured was also missing.[25] One detail not registered was the grade or level of play, although it could occasionally be read in the text file that the athlete competed in a national championship.

The results from comparing the injury claim data with desirable information in the Consensus statements [6 10-13] show that the insurance database could be useful in research on sport injuries. However, missing data on personal information such as height, body mass, dominant leg/arm, position of play, and return to play are important in some research. Information about return to play is often used to calculate the absence of sporting activity and hence the severity

For peer review only

of the injury.[5] In the insurance data, injury type and level of permanent medical disability are two methods of grading injury severity. The definition of medical disability is set by "Insurance Sweden" (the industry organization for insurance companies in Sweden). http://www.svenskforsakring.se/Global/Invaliditetsintyg/Medicinsk_invaliditet_skador120911.pdf

A recent published consensus statement for injury surveillance in Athletics is based on previous consensus statements [6 10-13] on details of athlete baseline and recordable incident/injury information. It also reflects more profound information on the specific issues facing Athletics at international and domestic levels.[34]

Prospective data collection procedure is considered to be the most appropriate method for sport injury surveillance.[6 10-13] When using insurance claims data both retrospectively and prospectively study design, can be used depending on the starting point of the study. Though, some of the claims may be reported to the insurance company retrospectively since the athlete can report an injury up to three years after the accident or in rare cases at the time when the athlete gained knowledge that injury may be reported to the insurance company (up to ten year after injury). In the present study where the data was retrospectively analyzed, injuries in the database were reported, in median 16 days from the day they occurred.

Van Mechelen suggests that an adequate sports injury surveillance system should be sensitive enough to answer "how many, how often, how long, and serious?".[35] If these questions are to be answered on a national level, where the magnitude of sports injury problems are investigated by comparing incidence rates between various sports, or detecting trends and changes over time, a system that can detect large numbers of cases is needed.[35] The system should be as simple and unambiguous as possible and should at least contain a universally applicable definition of sports injury, of sports participation and consensus on population at risk and time at risk.[35] The insurance data in this study are contained in a national register which covers all athletes in more than 80% of all sport federations, and includes, for example, sports with many athletes such as football, ice hockey, handball, floorball, golf, material arts and motor sports.

Lack of full information within some items both compared with ASIDD and the Consensus statements suggests that the insurance data could be improved upon in the future with the collection of additional details and standardizations of data variables. Some of these changes are implemented in the present insurance database as the result of this study. For example, by using headlines in the database, the terminology regarding injury mechanisms and injury

For peer review only

circumstances, match/training, surface and free text around the injury have been standardized based on desirable sport injury information in the literature. [6 10-13 25] This, enhance the possibility for future prospective studies of purpose-design surveillance at national level, with a large number of athletes and long follow up time.

Missing desirable information in the insurance data, including total number of athletes in each sport and exposure to sport, could be a weakness when performing epidemiological studies on sport injuries.[5 36] In further studies using data from the insurance company, assessment of sports injury incidence rate (the number of new sports injuries / population at risk over time) or sports injury prevalence (the proportion of injured athlete in the population at risk at any one time) may be calculated by using information from the specific sports federation regarding the number of participants and licensed athletes in each sport.[35 37] When conducting studies on exposure related incidence of injury an estimation of exposure to sport must be performed, for example by using estimated information from the specific sports federations regarding time spent on training and competition for each age group and standard/level of sport.

Despite the quality of the sports injury surveillance system, one will always be confronted with some sort of bias, recall bias, over- or underestimation of sports participation, incomplete responses, non-response, invalid injury description etc.[35] The insurance data collection is structured and routinely kept in a systematic manner, and the insurance company works to keep the data accurate and truthful. The database population consists of athletes who *report* an injury to the insurance company and therefore the data it contains likely underestimates the number of injuries. A study from 2005 on anterior cruciate ligament injuries in football, floorball, handball and ice hockey used the same insurance data as the present study and compared it to two medical databases to determine recording rate reliability.[18] The result showed that 74% of all injuries sustained were recorded in the insurance database.[18] Roos et al (1995) also studying the same database estimated that 98% of the football injuries were registered within seven years.[38] The amount of financial compensation for each claim from the insurance company may influence the rate of injury registration. More severe injuries that require medical treatment or may result in a permanent medical disability are more likely to be reported, as well as dental injuries where treatment is expensive. The athlete can report the injury within three years after the accident, and medical disabilities are assessed in one to two years after the accident occurred. This may result in a delay of recording in the database which must be considered when analyzing the data.[38]

For peer review only

Despite these weaknesses, the data are important for the detection of serious injuries and injury mechanisms in different sports at national level. Injury data, such as incidence and severity of injury can be compared between sports, gender and ages and be followed over time, for example in longitudinal studies of injury prevention measures at a national level.

CONCLUSION

Before preventative actions can be suggested, sports injury epidemiology, risk factors and injury mechanisms need to be compiled. Since a comprehensive national database from a Swedish insurance company contains most of the desirable information in sport injury research, and further improvements to the database information have been implemented, this database now has the potential to be a base for research on acute (accidental) injuries in organized sports at a national level. It could also constitute a foundation for assessment of sport injury prevention at a national level.

Funding

This work was supported by Folksam, a Swedish insurance company

Contributor ship statement

The corresponding author and co-authors have all substantial contributed to the research and to the final document by intellectual content, conception and design, analysis and interpretation of data as well as drafting and critical revision of the manuscript.

Data sharing statement

No additional data available.

Competing Interests

None declared

REFERENCES

1. Caine D, Caine C, Maffulli N. Incidence and distribution of pediatric sport-related injuries. Clin J Sport Med 2006;**16**(6):500-13 doi: 10.1097/01.jsm.0000251181.36582.a0[published Online First: Epub Date]|.
2. Conn JM, Annest JL, Gilchrist J. Sports and recreation related injury episodes in the US population, 1997-99. Inj Prev 2003;**9**(2):117-23
3. (EuroSafe) EAfIPaSP. Injuries in the European Union. Summary of injury statisticsfor the years 2008-2010. 2013(4)
4. Knowles SB, Marshall SW, Bowling JM, et al. A prospective study of injury incidence among North Carolina high school athletes. Am J Epidemiol 2006;**164**(12):1209-21 doi: 10.1093/aje/kwj337[published Online First: Epub Date]|.

For peer review only

5. van Mechelen W, Hlobil H, Kemper HC. Incidence, severity, aetiology and prevention of sports injuries. A review of concepts. Sports Med 1992;**14**(2):82-99
6. Fuller CW, Ekstrand J, Junge A, et al. Consensus statement on injury definitions and data collection procedures in studies of football (soccer) injuries. Clin J Sport Med 2006;**16**(2):97-106
7. Finch C. A new framework for research leading to sports injury prevention. J Sci Med Sport 2006;**9**(1-2):3-9; discussion 10 doi: 10.1016/j.jsams.2006.02.009[published Online First: Epub Date]|.
8. Junge A, Engebretsen L, Alonso JM, et al. Injury surveillance in multi-sport events: the International Olympic Committee approach. Br J Sports Med 2008;**42**(6):413-21 doi: 10.1136/bjsm.2008.046631[published Online First: Epub Date]|.
9. Hammond LE, Lilley J, Ribbans WJ. Coding sports injury surveillance data: has version 10 of the Orchard Sports Injury Classification System improved the classification of sports medicine diagnoses? Br J Sports Med 2009;**43**(7):498-502 doi: 10.1136/bjsm.2008.051979[published Online First: Epub Date]|.
10. Turner M, Fuller CW, Egan D, et al. European consensus on epidemiological studies of injuries in the thoroughbred horse racing industry. Br J Sports Med 2012;**46**(10):704-8 doi: 10.1136/bjsports-2011-090312[published Online First: Epub Date]|.
11. Fuller CW, Molloy MG, Bagate C, et al. Consensus statement on injury definitions and data collection procedures for studies of injuries in rugby union. Clin J Sport Med 2007;**17**(3):177-81 doi: 10.1097/JSM.0b013e31803220b3[published Online First: Epub Date]|.
12. Pluim BM, Fuller CW, Batt ME, et al. Consensus statement on epidemiological studies of medical conditions in tennis, April 2009. Br J Sports Med 2009;**43**(12):893-7 doi: 10.1136/bjsm.2009.064915[published Online First: Epub Date]|.
13. Orchard J, Newman D, Stretch R, Frost W, Mansingh A, Leipus A. Methods for injury surveillance in international cricket. J Sci Med Sport 2005;**8**(1):1-14
14. Nelson NG, McKenzie LB. Mountain biking-related injuries treated in emergency departments in the United States, 1994-2007. Am J Sports Med 2011;**39**(2):404-9 doi: 10.1177/0363546510383478[published Online First: Epub Date]|.
15. de Loes M, Dahlstedt LJ, Thomee R. A 7-year study on risks and costs of knee injuries in male and female youth participants in 12 sports. Scand J Med Sci Sports 2000;**10**(2):90-7
16. Otago L, Peake J. The role of insurance data in setting priorities for netball injury prevention strategies. J Sci Med Sport 2007;**10**(2):105-9 doi: 10.1016/j.jsams.2006.05.016[published Online First: Epub Date]|.
17. Soderman K, Pietila T, Alfredson H, Werner S. Anterior cruciate ligament injuries in young females playing soccer at senior levels. Scand J Med Sci Sports 2002;**12**(2):65-8
18. Forssblad M, Weidenhielm L, Werner S. Knee surgery costs in football, floor ball, European team handball and ice hockey. Scand J Med Sci Sports 2005;**15**(1):43-7 doi: 10.1111/j.1600-0838.2004.00392.x[published Online First: Epub Date]|.
19. Westin M, Alricsson M, Werner S. Injury profile of competitive alpine skiers: a five-year cohort study. Knee Surg Sports Traumatol Arthrosc 2012;**20**(6):1175-81 doi: 10.1007/s00167-012-1921-x[published Online First: Epub Date]|.
20. Junge A, Dvorak J. Injury surveillance in the World Football Tournaments 1998-2012. Br J Sports Med 2013;**47**(12):782-8 doi: 10.1136/bjsports-2013-092205[published Online First: Epub Date]|.

For peer review only

21. Engebretsen L, Soligard T, Steffen K, et al. Sports injuries and illnesses during the London Summer Olympic Games 2012. Br J Sports Med 2013;**47**(7):407-14 doi: 10.1136/bjsports-2013-092380[published Online First: Epub Date]|.
22. Engebretsen L, Steffen K, Alonso JM, et al. Sports injuries and illnesses during the Winter Olympic Games 2010. Br J Sports Med 2010;**44**(11):772-80 doi: 10.1136/bjsm.2010.076992[published Online First: Epub Date]|.
23. Finch C. How useful are insurance claim data for sports injury prevention purposes? Inj Control Saf Promot. 2003/07/17 ed, 2003:181-3.
24. King DA, Hume PA, Milburn P, Gianotti S. Rugby league injuries in New Zealand: a review of 8 years of Accident Compensation Corporation injury entitlement claims and costs. Br J Sports Med 2009;**43**(8):595-602 doi: 10.1136/bjsm.2009.061481[published Online First: Epub Date]|.
25. Party TASIDW. Australian Sports Injury Data Dictionary. Guidelines for injury data collection and classification for the prevention and control of injury in sport and recreation., 1997.
26. Gavrielov-Yusim N, Friger M. Use of administrative medical databases in population-based research. J Epidemiol Community Health 2013 doi: 10.1136/jech-2013-202744[published Online First: Epub Date]|.
27. Kucera KL, Marshall SW, Bell DR, DiStefano MJ, Goerger CP, Oyama S. Validity of soccer injury data from the National Collegiate Athletic Association's Injury Surveillance System. J Athl Train 2011;**46**(5):489-99
28. Robinson TW, Corlette J, Collins CL, Comstock RD. Shoulder injuries among US high school athletes, 2005/2006-2011/2012. Pediatrics 2014;**133**(2):272-9 doi: 10.1542/peds.2013-2279[published Online First: Epub Date]|.
29. Finch CF. An overview of some definitional issues for sports injury surveillance. Sports Med 1997;**24**(3):157-63
30. Kujala UM, Taimela S, Antti-Poika I, Orava S, Tuominen R, Myllynen P. Acute injuries in soccer, ice hockey, volleyball, basketball, judo, and karate: analysis of national registry data. BMJ 1995;**311**(7018):1465-8
31. Badii M, Keen D, Yu S, Yassi A. Evaluation of a comprehensive integrated workplace-based program to reduce occupational musculoskeletal injury and its associated morbidity in a large hospital. Journal of occupational and environmental medicine / American College of Occupational and Environmental Medicine 2006;**48**(11):1159-65 doi: 10.1097/01.jom.0000243416.18334.c8[published Online First: Epub Date]|.
32. McCall BP, Horwitz IB, Taylor OA. Occupational eye injury and risk reduction: Kentucky workers' compensation claim analysis 1994-2003. Inj Prev 2009;**15**(3):176-82 doi: 10.1136/ip.2008.020024[published Online First: Epub Date]|.
33. Krafft M, Kullgren A, Lie A, Tingvall C. The use of seat belts in cars with smart seat belt reminders--results of an observational study. Traffic Inj Prev 2006;**7**(2):125-9 doi: 10.1080/15389580500509278[published Online First: Epub Date]|.
34. Timpka T, Alonso JM, Jacobsson J, et al. Injury and illness definitions and data collection procedures for use in epidemiological studies in Athletics (track and field): consensus statement. Br J Sports Med 2014;**48**(7):483-90 doi: 10.1136/bjsports-2013-093241[published Online First: Epub Date]|.
35. van Mechelen W. Sports injury surveillance systems. 'One size fits all'? Sports Med 1997;**24**(3):164-8
36. de Loes M. Exposure data. Why are they needed? Sports Med 1997;**24**(3):172-5
37. Brooks JH, Fuller CW. The influence of methodological issues on the results and conclusions from epidemiological studies of sports injuries: illustrative examples. Sports Med 2006;**36**(6):459-72

For peer review only

38. Roos H, Ornell M, Gardsell P, Lohmander LS, Lindstrand A. Soccer after anterior cruciate ligament injury--an incompatible combination? A national survey of incidence and risk factors and a 7-year follow-up of 310 players. Acta orthopaedica Scandinavica 1995;**66**(2):107-12

For peer review only
